# Enhancing dementia risk screening with GAN-synthesized periodontal examination and general blood test data

**DOI:** 10.3389/fneur.2024.1379916

**Published:** 2024-08-14

**Authors:** Katsunori Oyama, Toshiki Isogai, Yohei Nakayama, Ryoki Kobayashi, Daisuke Kitano, Kenji Karako, Kaoru Sakatani

**Affiliations:** ^1^Department of Computer Science, College of Engineering, Nihon University, Koriyama, Japan; ^2^Graduate School of Computer Science, Nihon University, Koriyama, Japan; ^3^Research Institute of Oral Science, Nihon University School of Dentistry at Matsudo, Matsudo, Japan; ^4^Department of Periodontology, Nihon University School of Dentistry at Matsudo, Matsudo, Japan; ^5^Department of Infection and Immunology, Nihon University School of Dentistry at Matsudo, Matsudo, Japan; ^6^Division of Cardiology, Department of Medicine, Nihon University School of Medicine, Itabashi, Japan; ^7^Department of Human and Engineered Environmental Studies, Graduate School of Frontier Sciences, The University of Tokyo, Kashiwa, Japan; ^8^Institute of Gerontology, The University of Tokyo, Bunkyo, Japan

**Keywords:** blood test, periodontal examination, deep learning, generative adversarial networks, cognitive function

## Abstract

**Introduction:**

This study aimed to investigate the effectiveness of data augmentation to improve dementia risk prediction using machine learning models. Recent studies have shown that basic blood tests are cost-effective in predicting cognitive function. However, developing models that address various conditions poses challenges due to constraints associated with blood test results and cognitive assessments, including high costs, limited sample sizes, and missing data from tests not performed in certain facilities. Despite being often limited by small sample sizes, periodontal examination data have also emerged as a cost-effective screening tool.

**Methods:**

To address these challenges, this study explored the effectiveness of data augmentation using the Synthetic Minority Over-sampling Technique for Regression with Gaussian noise (SMOGN), a Generative Adversarial Network (GAN), and a Conditional Tabular GAN (CTGAN) on periodontal examination and blood test data. The datasets included parameters such as cognitive assessment results from the Mini-Mental State Examination (MMSE), demographic characteristics, periodontal examination data, and blood test results. Linear regression models, random forests, and deep neural networks were used to evaluate the effectiveness of the synthesized data.

**Results:**

This study used measured data from 108 participants and the synthesized data generated from the measured data. External validity was evaluated using a different dataset of 41 participants with missing items. The results suggested that normal GANs have the advantage of investigating models in data diversity, whereas CTGANs preserve the data structure and linear relationships in tabular data from the measured data, which drastically improves linear regression models.

**Discussion:**

Importantly, by interpolating sparse areas in the distribution, such as age, the synthesized models maintained prediction accuracy for test data with extreme inputs. These findings suggest that GAN-synthesized data can effectively address regression problems and improve dementia risk prediction.

## 1 Introduction

The rapidly increasing older population has led to a rise in the prevalence of dementia, including Alzheimer’s disease (AD). The number of people living with dementia across the world is expected to increase from 55 million in 2019 to 139 million in 2050 (1). Accurate diagnosis remains complex because of the subtleties of mental status assessment and the similarity of AD to other types of dementia. Mild cognitive impairment (MCI), often a precursor to AD, is widely recognized as crucial for early detection and intervention.

Blood tests, which have recently been correlated with Mini-Mental State Examination (MMSE) scores (2, 3), have emerged as a promising tool for cognitive screening. However, because of a variety of factors, machine learning models trained on blood test data often face limitations in accurately predicting dementia risks. One significant challenge is heterogeneity in patient data, including a wide range of biomarkers and cognitive scores. This variability can lead to inconsistencies in model performance, especially when dealing with multifaceted diseases such as dementia. Additionally, standard analytical models often struggle with the sparse and imbalanced nature of medical datasets, which can result in overfitting or the underrepresentation of certain patient groups. To address these issues, there is a growing need for innovative approaches capable of effectively handling diverse and incomplete data while maintaining predictive accuracy and reliability.

In addition to blood tests, recent research has highlighted the potential role of periodontal examination data in dementia risk assessment. Some studies have suggested a close relationship between cognitive function, oral health, and systemic metabolic function in older adults, with the number of healthy teeth being a significant predictor (4). However, to the best of our knowledge, the combination of periodontal examination and blood test results have never been investigated for cost-effective and rapid screening of dementia risk. It is because the integration of periodontal examination data with blood tests still faces the obstacles, including the associated high costs, limited sample sizes, and missing data from unperformed tests, while periodontal health is increasingly recognized for its potential links with cognitive function, offering a promising avenue for early dementia detection.

Recent studies applying generative adversarial networks (GANs) for clinical applications, including diagnosis, prediction, and anomaly detection, can mostly be found in the field of medical imaging (5, 6). This is the first study to integrate periodontal examination and blood test data and to apply synthesized models from tabular data for dementia risk prediction, which aim to address the challenges of data scarcity and heterogeneity in medical datasets that often impede accurate dementia risk prediction. This approach represents a step forward for cost-effective and rapid screening methods in early-stage dementia risk assessment.

## 2 Methods

**Figure 1:**
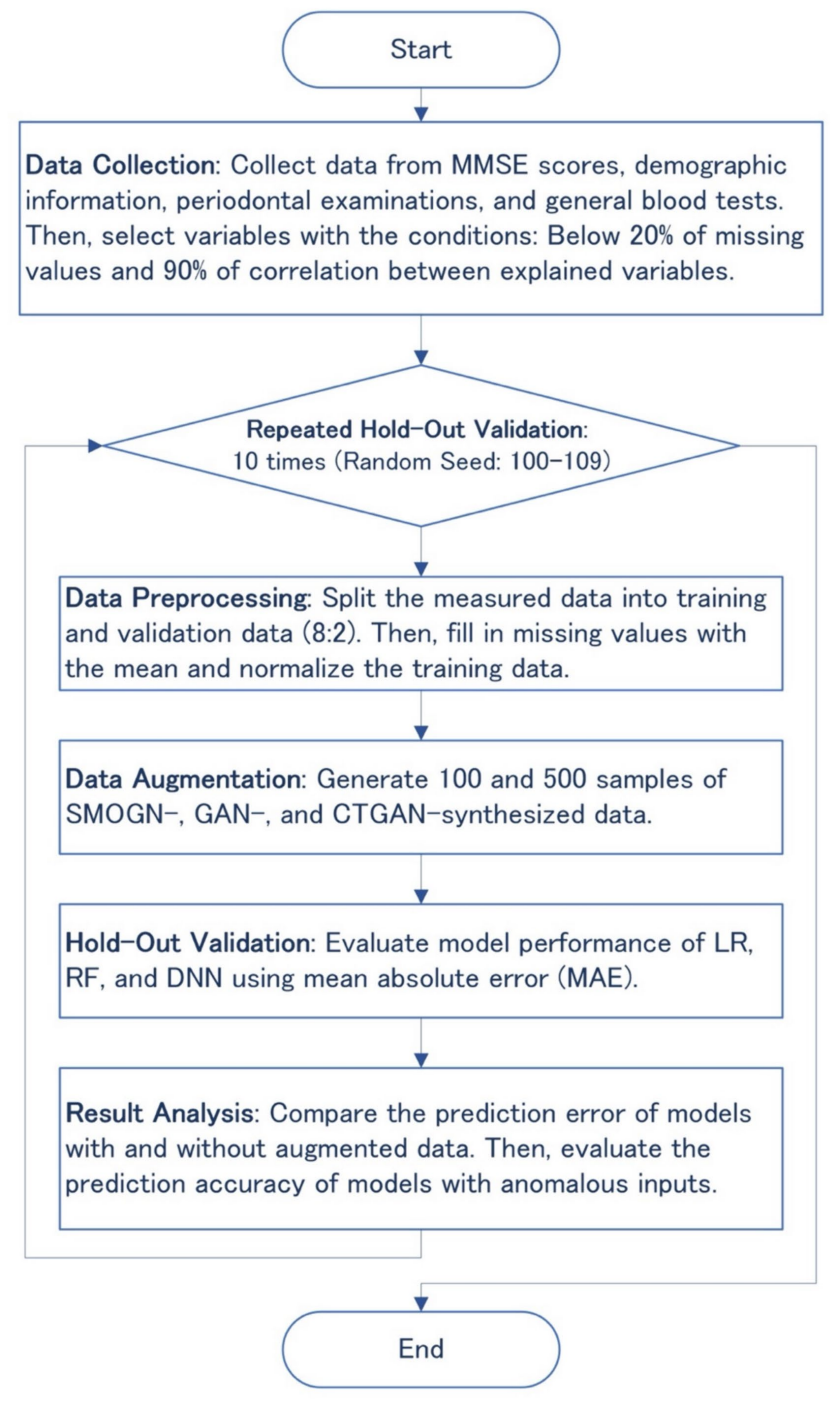
Flowchart of the overall methodology, including data collection, preprocessing, augmentation, hold-out validation, and result analysis.

Data augmentation techniques such as SMOGN, GAN, and CTGAN were applied to generate synthesized datasets after preprocessing the measured data to handle missing values, as shown in Figure 1. The synthesized datasets were then used for training three basic machine learning models: linear regression (LR), random forest (RF), and deep neural network (DNN). Prediction errors and the robustness of the synthesized models were evaluated through 10 repeated hold-out validation process with different random seeds to ensure the reliability and generalizability of the synthesized data and models.

### 2.1 Participants and measurements

We evaluated 108 individuals who were appointed for oral health assessments at Nihon University Itabashi Hospital (mean age ± standard deviation [SD], 69.4 ± 9.7 years; age range, 33–85 years). Written informed consent was obtained from all participants who agreed to additionally take MMSE and blood tests after receiving ethical approval from the Institutional Review Board (approval No.: RK-191210-3). Periodontal examinations, performed by a dentist in the research project, included assessments such as the number of remaining healthy teeth.

Regarding the external validity of the synthesized models, we used a dataset of 41 participants (28 males, 13 females, mean age ± SD, 69.7 ± 5.6 years) from a public health center in Koriyama, Fukushima, Japan, published in a preliminary study (4). The mean MMSE score was 26.7 ± 2.1. Compared with the measured data shown in Table 1, the external validation test dataset lacked thirteen of 27 items; however, the remaining 14 items were commonly available as predictor variables between the measured data and the external validation test dataset: age, sex, white blood cell count (WBC), hemoglobin (Hb), platelet count (Plt), aspartate aminotransferase (AST), alanine aminotransferase (ALT), total cholesterol (T-Cho), triglyceride (TG), blood urea nitrogen (BUN), creatinine (Cr), uric acid (UA), total protein (TP), and number of remaining teeth.

**Table 1:** Measured data (*N* = 108) with the parameters of Pearson’s correlation with MMSE scores and statistical difference between two MMSE groups (<28, ≥28).

Item		All (*N* = 108)	Pearson’s correlation	MMSE <28 (*n* = 45)	MMSE ≥28 (*n* = 63)	*p*-value
Response	MMSE	27.5 ± 2.4		25.2 ± 1.9	29.2 ± 0.8	**
Demographic data	Age (years)	69.4 ± 9.7	−0.32*	72.3 ± 8.4	67.3 ± 10	**
	Sex	M:88/F:20		M:51/F:12	M:37/F:8	
	Height (cm)	164.3 ± 0.1	0.19	1.63 ± 0.08	1.65 ± 0.08	*
	Weight (kg)	65.2 ± 11.2	0.11	63.6 ± 10.4	66.3 ± 11.6	
	sBP	128.7 ± 17.5	−0.08	129.1 ± 18.8	128.4 ± 16.6	
	dBP	75.2 ± 10.6	0	74.2 ± 10.5	75.9 ± 10.7	
	HR	73.8 ± 12.8	−0.07	74.8 ± 12.4	73.0 ± 13.0	
General blood test	WBC	5623.8 ± 1458.9	−0.04	5,650 ± 1464.4	5606.3 ± 1466.7	
	Hb	13.8 ± 1.4	0.08	13.6 ± 1.6	14.0 ± 1.2	
	Plt	21.1 ± 6.4	−0.04	20.8 ± 6.3	21.4 ± 6.5	
	AST	24.8 ± 8.9	−0.10	24.7 ± 9.2	24.8 ± 8.7	
	ALT	23.1 ± 14.1	−0.01	21.9 ± 13.6	24.0 ± 14.4	
	LDH	179.3 ± 30.7	−0.17	184.2 ± 37	175.9 ± 25.1	
	T-Cho	171.0 ± 33.8	0.07	171.3 ± 31.3	170.9 ± 35.5	
	HDL cholesterol	56.3 ± 15.2	−0.01	57.9 ± 16.8	55.3 ± 14.2	
	LDL cholesterol	85.5 ± 27.1	0.14	82.8 ± 24.1	87.2 ± 28.9	
	TG	144.6 ± 114.7	−0.04	149.5 ± 115.8	141.4 ± 114.9	
	BUN	17.5 ± 5.3	−0.22*	19.0 ± 6.3	16.4 ± 4.1	**
	Cr	0.9 ± 0.3	−0.04	1.0 ± 0.4	0.9 ± 0.3	
	UA	5.5 ± 1.3	0.11	5.3 ± 1.3	5.6 ± 1.3	
	TP	7.1 ± 0.5	0.04	7.0 ± 0.6	7.1 ± 0.4	
	HbA1c	6.1 ± 0.7	−0.05	6.1 ± 0.9	6.1 ± 0.6	
Periodontal examination	No. of remaining teeth	22.3 ± 6.8	0.18	21.1 ± 7.6	23.0 ± 6.2	
	Ave. PD	2.7 ± 0.5	−0.08	2.7 ± 0.4	2.7 ± 0.6	
	Ave. CAL	3.9 ± 1.1	−0.12	3.9 ± 1.1	3.8 ± 1.1	
	PISA	221.2 ± 215.8	0.05	197.8 ± 203.1	236.7 ± 224.1	
	PESA	1108.1 ± 372.6	0.17	1036.4 ± 377.5	1155.4 ± 364.6	*

MMSE, Mini-Mental State Examination; WBC, white blood cell count; Hb, hemoglobin; Plt, platelet count; AST, aspartate aminotransferase; T-Cho, total cholesterol; HDL, high-density lipoprotein; LDL, low-density lipoprotein; TG, triglycerides; BUN, blood urea nitrogen; UA, uric acid; Ave. PD, average probing depth; Ave. CAL, average clinical attachment level; PISA, periodontal inflammatory surface area; PESA, periodontal epithelial surface area.*p < 0.05, **p < 0.01.

### 2.2 Data augmentation

GANs were used to generate synthesized data from our dataset with combinations of sample sizes (100 and 500) and learning epochs (300, 1,000, 3,000, 5,000), while SMOGN served as the base model, effectively balancing the data distribution for oversampling in regression problems. For more details, the learning parameters of SMOGN, GAN, and CTGAN are listed in Supplementary Table S1.

#### 2.2.1 Synthesizer 1—synthetic minority over-sampling technique for regression with Gaussian noise

To address data imbalances, we applied SMOGN, which enhances minority data representation by generating synthetic samples with a Gaussian noise model (7). This technique ensures that the dataset remains representative of the original distribution for training unbiased models.

#### 2.2.2 Synthesizer 2—GAN

Our standard GAN were implemented using the TensorFlow 2 library. GANs operate using a pair of neural networks—the Generator and the Discriminator—which are trained concurrently through a competitive process. The Generator creates data from random noise, learning to make it indistinguishable from measured data. Conversely, the Discriminator learns to distinguish accurately whether the data presented is generated or real (8). The standard GAN in this study features a Generator and Discriminator, each with three hidden layers. These layers were configured with 256, 128, and 64 units for the Generator, and 128, 64, and 32 units for the Discriminator. We opted for the leaky rectified linear unit activation function to maintain gradient flow during training as a standard model of GAN, with each layer followed by batch normalization. The model was trained using a batch size of 16 and a learning rate of 0.0001 with the ADAM optimizer, which was selected based on preliminary experiments to optimize convergence.

#### 2.2.3 Synthesizer 3—conditional tabular GAN

In addition to the standard GAN, a CTGAN (9) was utilized because of its proficiency in synthesizing tabular data while preserving conditional distributions, which is particularly effective if a medical dataset needs to preserve specific statistical characteristics. The CTGAN open source library in the Synthetic Data Vault project is adept at capturing complex relationships between variables in a table format, making it particularly suitable for medical datasets, which often contain a mix of categorical and continuous features and require the statistical properties of the original data to be retained. In our application, CTGAN was applied to generate synthetic yet realistic and representative patient data, thereby enhancing the training process by providing a richer and more diverse set of samples for improving the generalization capabilities of our dementia risk prediction models. The model was trained with 5,000 epochs, a batch size of 500, and a learning rate of 0.0002 to achieve optimized convergence.

### 2.3 Machine learning models for data analysis

We employed LR, RF, and DNN models to estimate MMSE scores using the measured and synthesized data. The LR served as a baseline for comparison because of its interpretability and previous applications in AD research. RF, which is known for its ability to handle nonlinear data and robustness to noise, was included to assess its performance in our context. The DNNs, which were constructed using Tensor Flow 2, consisted of four hidden layers designed to capture intricate relationships. For more details, the learning parameters of RF and DNN are listed as Supplementary Table S2.

#### 2.3.1 LR

We implemented ordinary least-squares regression as our baseline model because of its interpretability and established use in AD research. This method provides a clear and straightforward way to analyze the linear relationship between predictor variables (e.g., blood test results, periodontal examination data) and MMSE scores.

#### 2.3.2 RF

Recognized for its ability to handle nonlinear relationships and robustness to noise, the RF model was constructed with 300 trees and a maximum depth of 10 using the scikit-learn library. This approach enhances predictive performance and helps control overfitting. The suitability of RF for AD research is supported by its success in similar applications (10).

#### 2.3.3 DNN

The neural network architecture included 19 input neurons, representing demographic, blood test, and periodontal examination data. Four hidden layers with descending neuron counts (256, 128, and 64) were used to process effectively the inputs as reported. We employed the scaled exponential linear unit activation function, which normalizes input signals to improve the training efficiency and stability. Batch normalization was applied after each layer. The network was trained using a batch size of 8 and a learning rate of 0.001 with the ADAM optimizer. These hyperparameters were optimized using a grid search to ensure stable model convergence.

## 3 Results

### 3.1 Statistical analysis of measured data

We systematically assessed cognitive function using the MMSE, complemented by demographic and blood test data, to explore correlations with periodontal health indicators, as shown in Table 1. Given the exploratory nature of our study, we reported unadjusted *p*-values to highlight potential associations. With an average MMSE score of 27.5 ± 2.4, the participants were categorized for further analysis into two groups based on MMSE scores: those with scores <28, which are indicative of possible MCI, and those with scores ≥28, which are considered within the normal cognitive range. The MMSE cut-off score of 27/28 is frequently employed in studies focusing on early detection of cognitive decline among older adults (11). Independent *t*-tests showed differences in the mean values for age, height, BUN, and PESA between these groups, suggesting their potential influence on cognitive status as determined by the MMSE (*p* < 0.01 for age and BUN; *p* < 0.05 for height and PESA).

The blood test items selected in Table 1 are commonly measured parameters in practice, and the periodontal examination items include PISA, PESA, and related computational metrics. For the subsequent machine learning analyses, a total of 27 variables were selected. These were chosen by having <20% missing values and ensuring a correlation coefficient < 0.9 between items to check for multicollinearity.

### 3.2 Results of data augmentation

For conducting the repeated hold-out validations, we employed SMOGN, GAN, and CTGAN to generate 10 sets of synthesized data from the measured dataset (Table 2). Both GAN and CTGAN were utilized for addition to the measured data for a comparison between them. The Kolmogorov–Smirnov (KS) complement scores indicated that SMOGN and CTGAN replicated the original data distribution more closely than GAN, especially for the MMSE score distribution in which CTGAN reached the highest score of 0.88 ± 0.04. It is noteworthy that GAN extensively extrapolates both in MMSE score and Age distributions around the minimum score (Min.) of the measured data, whereas SMOGN and CTGAN maintain the measured data distribution.

**Table 2:** Augmentation results: 10 sets of synthesized data for repeated hold-out validation.

		Measured data (no augmentation)	SMOGN	GAN	CTGAN
Sample size of training data		86.4 ± 0.5			
Size of output synthesized data		–	108.3 ± 8.7	100 or 500	100 or 500
MMSE score	Min.	21 ± 0	20.9 ± 0.0	14.4 ± 2.4	21.0 ± 0.0
	Mean	27.4 ± 0.1	27.1 ± 0.2	27.2 ± 0.3	26.7 ± 0.4
	KS complement score	1	0.83 ± 0.01	0.75 ± 0.07	0.88 ± 0.04
Age	Min.	33.0 ± 8.0	33.0 ± 8.0	0.0 ± 16.7	33.0 ± 8.0
	Mean	68.5 ± 0.6	69.1 ± 0.6	66.5 ± 1.5	65.8 ± 2.2
	Max.	85.0 ± 0.0	85.0 ± 0.1	85.0 ± 8.7	85.0 ± 0.0
	KS complement score	1	0.89 ± 0.02	0.83 ± 0.06	0.86 ± 0.03
	Similarity of Pearson’s correlation coefficients	1	0.91 ± 0.02	0.79 ± 0.06	0.80 ± 0.07

SMOGN, synthetic minority over-sampling technique for regression with Gaussian noise; GAN, generative adversarial network; CTGAN, conditional tabular GAN.

**Figure 2:**
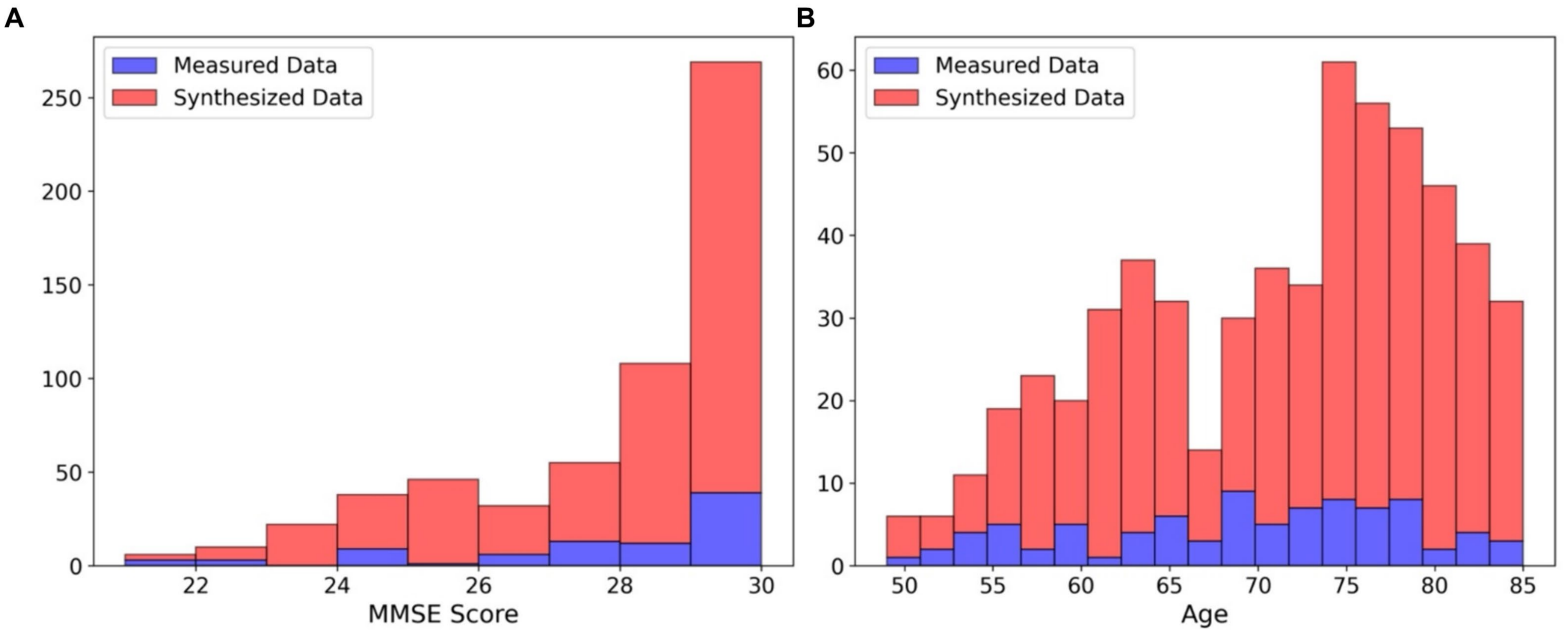
Distributions of MMSE scores and age. **(A)** Distribution of MMSE scores: measured data (blue) and synthesized data (red). **(B)** Distribution of age: measured data (blue) and synthesized data (red).

To understand how CTGAN interpolates the measured data, distributions of MMSE score and Age are compared as shown in Figure 2. In terms of the age distribution, CTGAN-synthesized data are closer to the measured data with a correlation of −0.27 ± 0.01, and the addition of CTGAN-synthesized data to the measured data only changes the correlation to MMSE scores within 0.05. Age distribution of the synthesized data also is closer to that of the measured data.

### 3.3 Results of hold-out validation

The LR, RF, and DNN models trained on measured data without periodontal examination items exhibited mean absolute errors (MAEs) of 2.10 ± 0.21, 1.95 ± 0.22, and 2.30 ± 0.27, respectively, in repeated hold-out validation (Table 3). After including periodontal examination items in the models, LR showed an increase in prediction error due to a larger number of variables, while RF and DNN exhibited improvements with mean MAEs of 2.28 ± 0.33, 1.94 ± 0.21, and 2.18 ± 0.23, respectively.

**Table 3:** Prediction errors in repeated hold-out validation using measured and synthesized data with and without periodontal examination items: most improved MAEs are highlighted.

Training (measured + synthesized data)	Learning epochs for GAN	Without periodontal examination items			With all available inputs		
		LR	RF	DNN	LR	RF	DNN
Measured data (*N* = 86) only		2.10 ± 0.21	1.95 ± 0.22	2.30 ± 0.27	2.28 ± 0.33	1.94 ± 0.21	2.18 ± 0.23
+ SMOGN (*N* = 75.4 ± 4.7)		2.19 ± 0.24	1.95 ± 0.17	2.27 ± 0.30	2.37 ± 0.31	1.94 ± 0.16	2.24 ± 0.27
+ GAN (*N* = 100)	300	2.08 ± 0.36	1.97 ± 0.28	2.27 ± 0.28	2.15 ± 0.37	1.96 ± 0.30	2.25 ± 0.34
	1,000	2.09 ± 0.23	1.98 ± 0.23	2.18 ± 0.26	2.18 ± 0.31	1.97 ± 0.22	2.02 ± 0.23
	3,000	2.12 ± 0.22	2.02 ± 0.28	2.26 ± 0.19	2.16 ± 0.24	1.98 ± 0.25	2.11 ± 0.25
	5,000	2.13 ± 0.13	2.04 ± 0.22	2.19 ± 0.27	2.23 ± 0.24	2.02 ± 0.24	2.19 ± 0.21
+ GAN (*N* = 500)	300	2.19 ± 0.43	1.96 ± 0.35	2.15 ± 0.43	2.25 ± 0.43	1.96 ± 0.35	2.09 ± 0.32
	1,000	2.18 ± 0.31	2.01 ± 0.26	2.25 ± 0.26	2.21 ± 0.38	1.98 ± 0.23	2.04 ± 0.27
	3,000	2.09 ± 0.24	2.06 ± 0.32	2.32 ± 0.28	2.08 ± 0.22	2.04 ± 0.28	2.20 ± 0.25
	5,000	2.19 ± 0.14	2.10 ± 0.24	2.17 ± 0.33	2.22 ± 0.23	2.07 ± 0.24	2.18 ± 0.28
+ CTGAN (*N* = 100)	300	2.05 ± 0.24	2.10 ± 0.30	2.20 ± 0.23	2.08 ± 0.27	2.09 ± 0.33	2.19 ± 0.20
	1,000	2.09 ± 0.16	2.09 ± 0.20	2.35 ± 0.16	2.10 ± 0.22	2.03 ± 0.21	2.28 ± 0.32
	3,000	1.99 ± 0.23	1.96 ± 0.21	2.33 ± 0.15	1.97 ± 0.25	1.95 ± 0.24	2.12 ± 0.26
	5,000	2.02 ± 0.24	2.02 ± 0.30	2.35 ± 0.38	2.06 ± 0.29	1.99 ± 0.29	2.40 ± 0.29
+ CTGAN (*N* = 500)	300	2.13 ± 0.30	2.25 ± 0.37	2.30 ± 0.24	2.13 ± 0.29	2.28 ± 0.38	2.20 ± 0.23
	1,000	2.14 ± 0.17	2.16 ± 0.27	2.31 ± 0.24	2.12 ± 0.17	2.14 ± 0.27	2.22 ± 0.25
	3,000	1.95 ± 0.27	2.05 ± 0.27	2.21 ± 0.26	1.96 ± 0.34	2.03 ± 0.28	2.12 ± 0.30
	5,000	**1.97 ± 0.24**	2.02 ± 0.26	2.19 ± 0.32	**1.96 ± 0.27**	2.00 ± 0.26	2.15 ± 0.30

LR, Linear Regression model; RF, Random Forest model; DNN, Deep Neural Network model.

**Figure 3:**
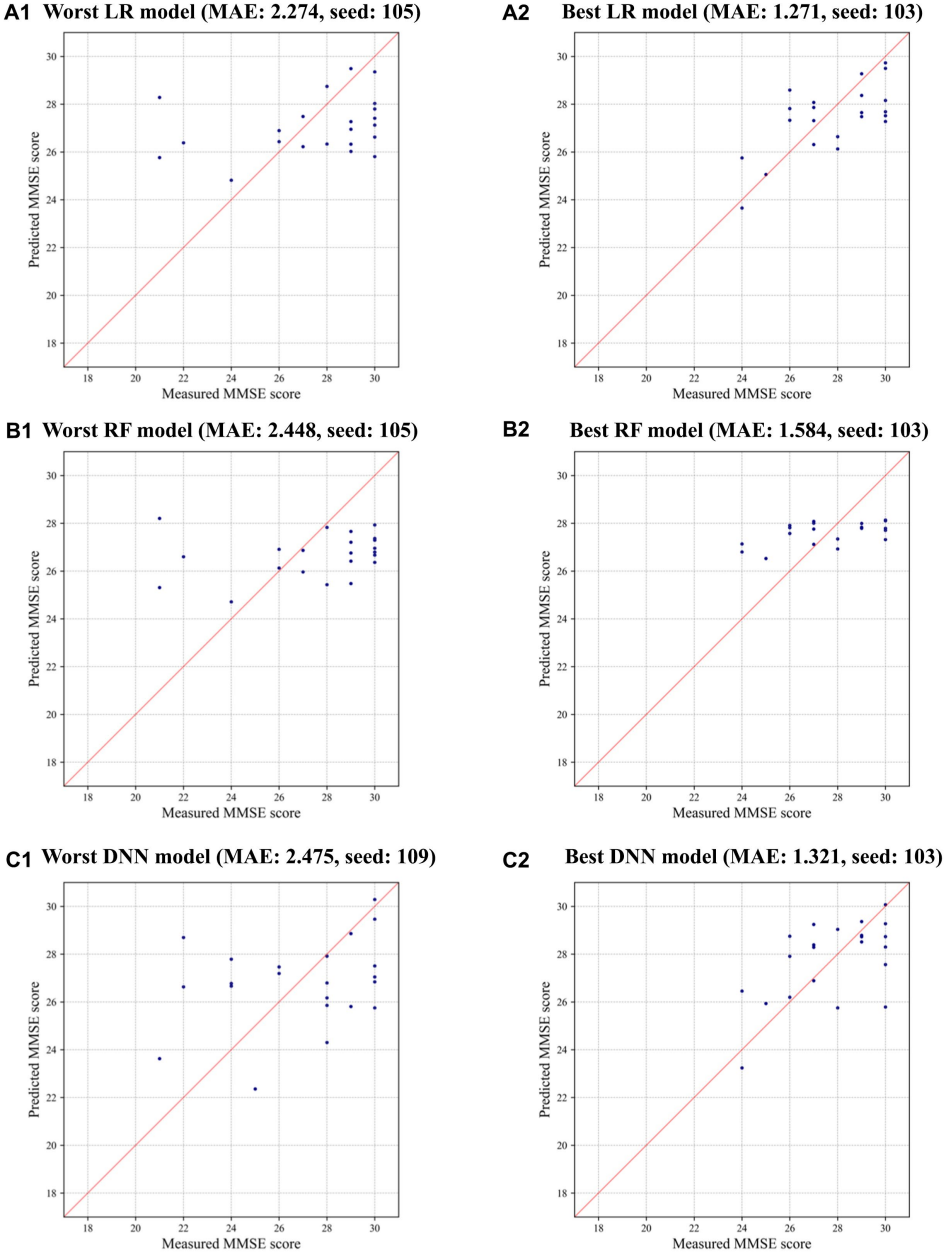
Measured and predicted scores in the worst and best models using CTGAN-synthesized data: **(A)** LR, **(B)** RF, and **(C)** DNN.

Interestingly, LR achieved the most significant improvement in prediction errors compared to RF and DNN when CTGAN-synthesized data (*N* = 500) were added to the measured data, as highlighted in Table 3. This result suggests that LR effectively utilizes CTGAN-synthesized data for dementia risk predictions, which outperforms RF and DNN models under these conditions. Figure 3 illustrate that, compared with the results of the worst models, CTGAN with the certain seed number drastically improved all LR, RF, and DNN models by showing proportional measured and predicted MMSE scores, while the models with a poor augmentation result can diffuse predicted MMSE scores.

Next, the mean ± SD of standard coefficients in the LR models were assessed to understand the variable importance of periodontal examination items (Table 4). While PESA originally showed higher importance with the measured data, its importance diminished when CTGAN-synthesized data were included. Instead, the average PD level became relatively important while the number of remaining teeth is known to be a good biomarker. To assess the dependency on age, we further analyzed the models by excluding this variable as listed in Table 4. BUN, Height, Weight, and PESA especially emerged as more influential variables in the LR models without Age.

**Table 4:** Standard coefficients in the LR models for comparison between measured data only (*N* = 108) and measured data + CTGAN-synthesized data (*N* = 108 + 500) in repeated hold-out validation: periodontal examination items are highlighted.

Variable (top 15)	LR using measured data (no augmentation)	Variable (top 15)	LR using measured data + CTGAN data	Variable (top 15)	LR using measured data + CTGAN data without age
**PESA**	1.33 ± 0.97	Age	−0.42 ± 0.15	Sex	0.35 ± 0.20
**RemainingTooth**	−0.91 ± 0.78	Sex	0.33 ± 0.19	BUN	−0.31 ± 0.10
BUN	−0.73 ± 0.19	BUN	−0.24 ± 0.09	Weight	0.22 ± 0.10
**AvePD**	−0.72 ± 0.47	**AvePD**	−0.18 ± 0.13	Height	0.22 ± 0.16
Sex	0.68 ± 0.19	Weight	0.16 ± 0.10	Plt	−0.15 ± 0.16
Age	−0.60 ± 0.12	Height	0.16 ± 0.15	**AvePD**	−0.15 ± 0.14
dBP	−0.38 ± 0.07	Plt	−0.16 ± 0.16	LDH	−0.14 ± 0.11
**PISA**	−0.37 ± 0.38	LDH	−0.10 ± 0.10	LDL	0.12 ± 0.15
Cr	0.37 ± 0.25	HR	−0.10 ± 0.11	HR	−0.11 ± 0.10
UA	0.36 ± 0.16	LDL	0.10 ± 0.14	**PESA**	0.10 ± 0.06
Height	0.34 ± 0.10	**RemainingTooth**	0.09 ± 0.09	**RemainingTooth**	0.10 ± 0.09
AST	−0.33 ± 0.22	ALT	−0.08 ± 0.10	Hb	0.10 ± 0.13
HDL	−0.29 ± 0.29	UA	0.08 ± 0.09	UA	0.08 ± 0.08
WBC	−0.23 ± 0.08	T-Cho	0.08 ± 0.11	**AveCAL**	−0.07 ± 0.16
TG	−0.22 ± 0.31	AST	−0.08 ± 0.11	T-Cho	0.07 ± 0.13

### 3.4 Robustness experiments

To evaluate model robustness against the insertion of anomalous data, validation tests were conducted with the input Age set to “0,” where Age is the important variable both for both original and synthesized models. In these cases, the models trained with measured data exhibited a significant increase in prediction errors. Specifically, LR, as a linear model, showed heightened sensitivity to anomalous values in both the measured and synthesized datasets. By contrast, the GAN-and CTGAN-synthesized models demonstrated stable MAEs as detailed in Table 5. These results suggest that handling diverseness in the data distribution during the augmentation process may be key to addressing challenges prevalent in medical datasets, such as imbalanced data and missing values.

**Table 5:** Prediction errors in repeated hold-out validation with the anomalous inputs by replacing test data: age = 0.

Training (measured + synthesized data with the anomalous inputs: **age = 0**)	LR	RF	DNN
Measured data (*N* = 86) only	4.66 ± 1.10	1.98 ± 0.24	2.58 ± 0.55
+ SMOGN (*N* = 75.4 ± 4.7)	5.05 ± 1.28	2.02 ± 0.19	2.33 ± 0.39
+ GAN (*N* = 500)	3.30 ± 1.77	2.00 ± 0.25	2.24 ± 0.44
+ CTGAN (*N* = 500)	3.40 ± 0.89	2.02 ± 0.28	2.10 ± 0.46

### 3.5 External validity

We also evaluated the prediction errors using the external validation test dataset (Table 6). CTGAN-synthesized LR exhibited a significant decrease in MAEs, to 1.55 ± 0.27 (18.8% improvements). Despite of the limited 14 common items, we confirmed that reasonable prediction error results are obtained using CTGAN-synthesized data.

**Table 6:** Prediction errors for the external validation test data (*N* = 41) using the 10 sets of measured and synthesized data: most improved MAEs are highlighted.

Training (measured + synthesized data)	LR	RF	DNN
Measured data (*N* = 86) only	1.91 ± 0.41	1.89 ± 0.10	1.87 ± 0.34
+ SMOGN (*N* = 75.4 ± 4.7)	2.13 ± 0.39	1.92 ± 0.22	2.03 ± 0.53
+ GAN (*N* = 500)	1.99 ± 0.83	1.74 ± 0.16	2.01 ± 0.50
+ CTGAN (*N* = 500)	**1.55 ± 0.27**	1.95 ± 0.28	1.90 ± 0.45

## 4 Discussion

### 4.1 Validity of periodontal examination and blood test data for dementia risk screening

The integration of periodontal examination and blood test data showed slight improvements. However, the average probing depth (PD) and the number of remaining teeth were identified as valuable biomarkers. This confirms the importance of tooth counts as a modifiable dementia risk factor (12) and the potential role of PD levels in dementia risk screening.

PESA can be useful for dementia risk screening; however, it is highly dependent on Age. PESA is calculated by summing the product of the remaining teeth and the probing depth (PD) level for each tooth, meaning the number of remaining teeth significantly affects the results. This suggests that further stratified analysis based on Age and the number of teeth may contribute to a better understanding of the importance of periodontal examination items if PESA is considered.

While the relationship between periodontitis and dementia is well established, other blood test items related to oxygen transport and nutrition did not show the expected variations with MMSE scores in our dataset. This could be attributed to the demographic characteristics of our participants, who were primarily patients undergoing oral health assessments and were less likely to exhibit symptoms typically associated with dementia, such as anemia, metabolic syndrome, and chronic inflammation.

Further research is needed to explore the interrelations between blood test results and periodontal disease. Although salivary levels of BUN and AST are known to be useful biomarkers for screening periodontal disease (13), whether blood test results are significantly influenced by periodontal disease remains unclear.

### 4.2 Effectiveness of synthesized medical tabular data for model performance

The synthesized data generated using GAN and CTGAN showed promising results, especially for improving the performance of the LR models. Interestingly, the inclusion of CTGAN-synthesized data (*N* = 500) significantly enhanced the predictive accuracy of the LR models more than RF and DNN models. This suggests that LR models are better suited for application of synthesized data in predicting dementia risk.

The findings also indicate that synthesized data can help handle the issue of limited sample sizes in medical research. Not only addition of CTGAN-synthesized data preserves the statistical properties of the original dataset, synthesized data are effective to improve the robustness and generalizability of machine learning models. This will be particularly important in the context of dementia risk prediction, where obtaining large and diverse datasets can be challenging.

We conclude that the potential of combining blood test results, periodontal examination data with synthesized data to improve dementia risk screening, and boot-strapping of synthesized data will be the key for successful models of dementia risk screening where data collection may be limited or expensive, on the other hand, it may always take some time to find the best result and some degree of automation is necessary for practical use.

### 4.3 Implications for dementia risk screening and limitations

The application of artificial intelligence (AI) and machine learning in health care, particularly in dementia research, represents a significant technological advancement. However, this comes with ethical responsibilities, including the protection of data privacy, informed patient consent, and the reliability of predictions. Collaborative efforts among data scientists, clinicians, and health-care professionals are more vital to ensure the responsible use of AI in medical practice than ever.

This study did have some limitations, including the initial dataset size and its representativeness. Previous studies have identified other crucial blood test components, such as Plt, glycated hemoglobin, albumin, and electrolytes (3, 14); however, we could not examine these components in the present study. Further research considering these elements could provide a more comprehensive understanding of dementia risk factors. Moreover, while the correlation between periodontitis and dementia is more evident, the causal relationships remain to be fully explained. In this sense, longitudinal data analysis to track changes over time and identify potential causal pathways can give good solutions.

This study also acknowledges that the training process for both GAN and CTGAN can be time-intensive and highly sensitive to learning parameters. Recent advancements in the field of GAN training may result in methodologies that can mitigate these challenges. The success of GANs in this study warrants their exploration in other health-care domains where data limitations are a significant barrier to innovation.

## 5 Conclusion

The results of this study confirm the potential of GANs in enhancing dementia risk prediction, particularly in settings with data limitations. The GAN- and CTGAN-synthesized models were able to maintain robustness against anomalies and outperform models trained with limited data. Future advancements in GAN methodologies could further revolutionize health-care technology and patient care in dementia and beyond.

## Data availability statement

The raw data supporting the conclusions of this article will be made available by the authors, without undue reservation.

## Ethics statement

The studies involving humans were approved by Nihon University Hospitals’ Joint Institutional Review Board. The studies were conducted in accordance with the local legislation and institutional requirements. The participants provided their written informed consent to participate in this study.

## Author contributions

KO: Conceptualization, Formal analysis, Funding acquisition, Investigation, Methodology, Project administration, Resources, Software, Supervision, Validation, Visualization, Writing – original draft, Writing – review & editing. TI: Data curation, Investigation, Writing – original draft. YN: Data curation, Funding acquisition, Investigation, Project administration, Writing – review & editing. RK: Data curation, Investigation, Writing – review & editing. DK: Data curation, Writing – review & editing. KK: Formal analysis, Writing – review & editing. KS: Supervision, Writing – review & editing.
