# Supplementary material for: Enhancing dementia risk screening with GAN-synthesized periodontal examination and general blood test data
Source: Front Neurol. 2024 Aug 14;15:1379916. doi: 10.3389/fneur.2024.1379916 (PMC11349569; doi:10.3389/fneur.2024.1379916)
Supplement: Supplementary file 1 [file Data_Sheet_1.pdf]

Enhancing Dementia Risk Screening with GAN-Synthesized Periodontal Examination and General Blood Test Data

Supplementary Material

Table S1. Learning Parameters for SMOGN, GAN, and CTGAN synthesizer models.

Synthesizer model	Parameter	Value
**SMOGN**	**Random Seeds**	10 seeds from 100 through 109
	**Sampling method**	balance
**GAN**	**Random Seeds**	10 seeds from 100 through 109
	**Batch size**	16
	**Learning Epochs**	Chosen from [300, 1000, 3000, 5000]
	**Learning rate (Generator)**	0.00005
	**Learning rate (Discriminator)**	0.00005
	**Hidden Layers (Generator)**	[256, 128, 64]
	**Hidden Layers (Discriminator)**	[128, 64, 32]
	**Activation Function**	Leaky ReLU
	**Optimizer**	ADAM
**CTGAN**	**Random Seeds**	10 seeds from 100 through 109
	**Batch size**	(Default) 500
	**Learning Epochs**	Chosen from [300, 1000, 3000, 5000]
	**Learning rate (Generator)**	(Default) 0.0002
	**Learning rate (Discriminator)**	(Default) 0.0002

Table S2. Learning Parameters for RF and DNN models.

Synthesizer model	Parameter	Value
**RF**	**Random Seeds**	10 seeds from 100 through 109
	**Num_of_estimators**	300
	**Max_depth**	10
	**Min_samples_split**	5
	**Min_samples_leaf**	4
	**Max_features**	sqrt
**DNN**	**Random Seeds**	10 seeds from 100 through 109
	**Learning Epochs**	[300, 1000, 3000, 5000]
	**Input Layer**	27 input nodes
	**Hidden Layers**	[256, 128, 64]
	**Batch Normalization**	On
	**L2 Regularizer**	0.001
	**Dropout Rate**	0.1
	**Output Layer**	1 output node
	**Optimizer**	Adam
	**Learning Rate**	0.001
	**Beta 1**	0.9
	**Beta 2**	0.999
	**Loss Function**	Mean Squared Error (MSE)
	**Metrics**	Mean Absolute Error (MAE)
	**Batch Size**	8
	**Validation Split**	20%

List S1. Data Augmentation Using SMOGN, GAN, and CTGAN

Introduction:

This script includes the process of generating synthetic data using three methods: SMOGN, Normal GAN, and CTGAN, to generate the synthesized data for comparing the augmentation results and the performance of dementia risk screening based on MMSE scores, demographic information, periodontal examinations, and general blood tests.

Variables and Columns:

COLUMNS_INDEX: ['n']
COLUMNS_RESPONSE: ['MMSE']
COLUMNS_CAT: ['Sex'] (Categorical variable)
LIST_COLUMNS_VARIABLES: Two lists of variable columns to be used in the validation.
One list includes periodontal examination data.
The other list excludes periodontal examination data.

Data Preparation:

(1) Loading Labeled Dataset:
The labeled dataset is loaded from the specified CSV file.
Rows with missing target variables (MMSE) are removed.
Missing values in numeric columns are filled with the mean of each column.
Indicator variables are added for columns with more than 10% missing values.

(2) Scaling Data:
The dataset is scaled using StandardScaler from the sklearn library to normalize the feature values.
The ColumnTransformer is used to apply the scaling only to numeric variables while keeping categorical and indicator variables unchanged.

Synthetic Data Generation:

(1) SMOGN:
Dynamic Relevance Function:
A function is defined to calculate quartiles and create relevance control points for SMOGN.
SMOGN Data Generation:
The smoter function from the smogn library is used to generate synthetic data based on the defined relevance function.
Numeric variables are inverse transformed and clipped to ensure reasonable values.
Categorical and indicator variables are restricted to values of 0 or 1.
Evaluation and Saving:
The quality of the synthesized data is evaluated and saved.
Synthetic data and quality reports are saved to CSV and text files, respectively.

(2) GAN:
Generator and Discriminator:
Classes for the generator and discriminator models are defined with respective architectures.
Custom loss functions for the generator and discriminator are defined.
GAN Training:
A function is defined to train the GAN models using the training dataset.
The generator is used to create synthetic data at different sizes.
Evaluation and Saving:
The quality of the synthesized data is evaluated and saved.
Synthetic data and quality reports are saved to CSV and text files, respectively.

(3) CTGAN:
The CTGANSynthesizer from the sdv library is used to create synthetic data.
The model is trained using the training dataset.
Evaluation and Saving:
The quality of the synthesized data is evaluated and saved.

Synthetic data and quality reports are saved to CSV and text files, respectively.

Code:

```
'''
Data augmentation using SMOGN, Normal GAN and CTGAN (Frontiers in Neurology)
May 2024
'''

import os
import time
from math import ceil
import numpy as np
import matplotlib.pyplot as plt
import pandas as pd

from sdv.single_table import CTGANSynthesizer
from sklearn.model_selection import train_test_split
from sklearn.preprocessing import StandardScaler
# from sklearn.preprocessing import OneHotEncoder
from sklearn.compose import ColumnTransformer

import tensorflow as tf
import keras.models
import keras.layers

from sdv.metadata import SingleTableMetadata
from sdv.evaluation.single_table import evaluate_quality
from sdv.evaluation.single_table import get_column_plot

pd.set_option('display.max_rows', None)

# # Target files and directories
filename = 'digitalhealth_data2023_0519_ZeroToNa.csv'
DATA_DIR = 'data'
BASE_DIRS = [os.path.join(DATA_DIR, 'RepeatedHoldOut_CTGAN_{}'.format(filename.rsplit('.', 1)[0])),
        os.path.join(DATA_DIR,
                'RepeatedHoldOut_CTGAN_without_PeriodontalExam_{}'.format(filename.rsplit('.', 1)[0]))]
BASE_DIR = BASE_DIRS[0]

# Basic parameters
INIT_RANDOM_SEED = 0
TEST_SIZE = 0.2

# Columns for the target data
COLUMNS_INDEX = ['n']
COLUMNS_CAT = ['Sex']  # Categorical variables to be included in COLUMNS_VARIABLES
COLUMNS_INDICATOR = []  # Indicator variables indicating missing values (not used in this version for analysis)
COLUMNS_RESPONSE = ['MMSE']  # Corresponds to a column y
LIST_COLUMNS_VARIABLES = [['Age', 'WBC', 'Hb', 'Plt', 'AST', 'ALT',
                'LDH',
                'T-Cho',
                'HDL', 'LDL',
                'TG', 'BUN', 'Cr', 'UA',
                'TP',
                'HbA1c',
                'Height', 'Weight',
                'sBP', 'dBP', 'HR',
```

```
                    'RemainingTooth',
                    'AvePD',
                    'AveCAL',
                    # 'BOP',
                    'PISA',
                    'PESA'
                    ] + COLUMNS_CAT,
                   ['Age', 'WBC', 'Hb', 'Plt', 'AST', 'ALT',
                    'LDH',
                    'T-Cho',
                    'HDL', 'LDL',
                    'TG', 'BUN', 'Cr', 'UA',
                    'TP',
                    'HbA1c',
                    'Height', 'Weight',
                    'sBP', 'dBP', 'HR'
                    ] + COLUMNS_CAT,
                   ]  # Corresponds to columns X
COLUMNS_VARIABLES = LIST_COLUMNS_VARIABLES[0]

# Hyper parameters for the Normal GAN and CTGAN
BATCH_SIZE = 16
GENERATOR_LEARNING_RATE = 1e-4
DISCRIMINATOR_LEARNING_RATE = 0.5e-4
LIST_GAN_EPOCHS = [300, 1000, 3000, 5000]
# LIST_GAN_SIZE = [100, 200, 500, 1000, 2000]
LIST_GAN_SIZE = [100, 200, 500, 2000]

for loop_index in range(0, 2):
    COLUMNS_VARIABLES = LIST_COLUMNS_VARIABLES[loop_index]
    BASE_DIR = BASE_DIRS[loop_index]
    if not os.path.exists(BASE_DIR):
        os.mkdir(BASE_DIR)


    # Loading and preprocessing dataset
    def load_and_preprocess_data(dataset_path, columns_index, columns_y, columns_x, columns_cat,
columns_indicator):
        df = pd.read_csv(dataset_path, encoding='shift-jis', index_col=columns_index)[
            columns_y + columns_x]

        # Remove rows with missing target variable
        df.dropna(subset=columns_y, axis=0, inplace=True)
        df.reset_index(inplace=True)

        # # Test: Remove rows with fewer than a specified number of non-missing values
        # df.dropna(thresh=20, axis=0, inplace=True)
        # df.reset_index(drop=True, inplace=True)

        # Add indicator variables for columns with more than 10% missing values
        for column in columns_x:
            missing_rate = df[column].isnull().mean()
            if missing_rate > 0.1:
                print(column + ": " + str(missing_rate * 100) + "% missing")
                df[column + '_isNA'] = df[column].isnull().astype(int)
                columns_indicator.append(column + '_isNA')

        # # Remove extreme outliers or abnormal inputs using 3σ rule
```

```
    # for column in df.columns:
    #     mean = df[column].mean()
    #     std = df[column].std()
    #     cutoff = 3 * std
    #     lower_limit = mean - cutoff
    #     upper_limit = mean + cutoff
    #     df = df[(df[column] >= lower_limit) & (df[column] <= upper_limit)]

    # Impute missing values in numeric variables with the mean
    columns_num = [col for col in columns_x if col not in columns_cat]
    df[columns_num] = df[columns_num].fillna(df[columns_num].mean(axis=0))

    return df


# Function for data standardization
def normalize_train_test_data(train_df, test_df, columns_index, columns_y, columns_x, columns_indicator,
                              columns_cat):
    # Exclude 'Sex' from the list of numeric variables
    columns_num = [col for col in columns_y + columns_x if col not in set(columns_index + columns_cat)]

    # Create a preprocessing pipeline
    preprocessor = ColumnTransformer(
        transformers=[
            ('num', StandardScaler(), columns_num)
        ],
        remainder='passthrough'
        # Standardize only numeric variables, keep categorical and indicator variables unchanged
    )

    # Standardize training data - Combine standardized columns with other columns to retain column order
    train_transformed = preprocessor.fit_transform(train_df)
    train_df_num = pd.DataFrame(train_transformed[:, :len(columns_num)], columns=columns_num)
    train_df_head = train_df[[col for col in train_df.columns if col in columns_index]].reset_index(drop=True)
    train_df_tail = train_df[[col for col in train_df.columns if
                              col not in columns_num and (
                                      col in columns_cat or col in columns_indicator)]].reset_index(
        drop=True)
    train_df_norm = pd.concat([train_df_head, train_df_num, train_df_tail], axis=1)

    # Standardize test data - Combine standardized columns with other columns to retain column order
    test_transformed = preprocessor.transform(test_df)
    test_df_num = pd.DataFrame(test_transformed[:, :len(columns_num)], columns=columns_num)
    test_df_head = train_df[[col for col in train_df.columns if col in columns_index]].reset_index(drop=True)
    test_df_tail = test_df[[col for col in test_df.columns if
                            col not in columns_num and (
                                    col in columns_cat or col in columns_indicator)]].reset_index(
        drop=True)
    test_df_norm = pd.concat([test_df_head, test_df_num, test_df_tail], axis=1)

    return train_df_norm, test_df_norm, preprocessor


# Load and initialize data
df = load_and_preprocess_data(os.path.join(DATA_DIR, filename), COLUMNS_INDEX,
COLUMNS_RESPONSE, COLUMNS_VARIABLES,
                              COLUMNS_CAT,
                              COLUMNS_INDICATOR)
```

```
'''
Definition of Normal GAN
'''


class Generator:
    def __init__(self, input_size=len(COLUMNS_RESPONSE + COLUMNS_VARIABLES +
COLUMNS_INDICATOR),
                 hidden_sizes=[256, 128, 64],
                 output_size=len(COLUMNS_RESPONSE + COLUMNS_VARIABLES + COLUMNS_INDICATOR)):
        self.l2 = tf.keras.regularizers.l2(0.0001)
        self.input = keras.layers.Input(shape=(input_size,))
        self.hidden_layers = []
        for size in hidden_sizes:
            self.hidden_layers.append(keras.layers.Dense(size, kernel_regularizer=self.l2))
        self.output = keras.layers.Dense(output_size, activation='linear')

    def build(self):
        x = self.input
        x = keras.layers.BatchNormalization()(x)
        x = keras.layers.LeakyReLU()(x)
        x = keras.layers.Dropout(0.2)(x)
        for layer in self.hidden_layers:
            x = keras.layers.BatchNormalization()(x)
            x = keras.layers.LeakyReLU()(layer(x))
        output = self.output(x)
        return keras.models.Model(inputs=self.input, outputs=output)


class Discriminator:
    def __init__(self, input_size=len(COLUMNS_RESPONSE + COLUMNS_VARIABLES +
COLUMNS_INDICATOR),
                 hidden_sizes=[128, 64, 32],
                 output_size=1):
        self.l2 = tf.keras.regularizers.l2(0.0001)
        self.input = keras.layers.Input(shape=(input_size,))
        self.hidden_layers = []
        for size in hidden_sizes:
            self.hidden_layers.append(keras.layers.Dense(size, kernel_regularizer=self.l2))
        self.output = keras.layers.Dense(output_size)

    def build(self):
        x = self.input
        x = keras.layers.LeakyReLU()(x)
        for layer in self.hidden_layers:
            x = keras.layers.Dropout(0.2)(x)
            x = keras.layers.LeakyReLU()(layer(x))
        output = self.output(x)
        return keras.models.Model(inputs=self.input, outputs=output)


# Loss functions and optimizers
cross_entropy = tf.keras.losses.BinaryCrossentropy(from_logits=True)
generator_optimizer = tf.keras.optimizers.Adam(learning_rate=GENERATOR_LEARNING_RATE)
discriminator_optimizer = tf.keras.optimizers.Adam(learning_rate=DISCRIMINATOR_LEARNING_RATE)
```

```
def discriminator_loss(real_output, fake_output):
    real_loss = cross_entropy(tf.ones_like(real_output) * 0.9, real_output)  # Label smoothing
    fake_loss = cross_entropy(tf.zeros_like(fake_output), fake_output)
    return real_loss + fake_loss


def generator_loss(fake_output):
    return cross_entropy(tf.ones_like(fake_output), fake_output)


def generate_noise(num_samples, columns_y=COLUMNS_RESPONSE, columns_x=COLUMNS_VARIABLES,
columns_cat=COLUMNS_CAT,
                   columns_ind=COLUMNS_INDICATOR):
    # Noise for numerical variables
    noise_num = tf.random.normal(
        [num_samples, len([col for col in columns_y + columns_x if col not in columns_cat])])

    # Noise for categorical variables
    noise_cat = tf.random.uniform([num_samples, len(columns_cat)], minval=0, maxval=2, dtype=tf.int32)
    noise_cat = tf.cast(noise_cat, tf.float32)

    # Noise for indicator variables
    noise_ind = tf.random.uniform([num_samples, len(columns_ind)], minval=0, maxval=2, dtype=tf.int32)
    noise_ind = tf.cast(noise_ind, tf.float32)
    # noise_ind = tf.zeros([num_samples, len(columns_indicator)], dtype=tf.float32)

    noise = tf.concat([noise_num, noise_cat, noise_ind], axis=1)

    return noise


def train_step(datas, generator, discriminator, generator_optimizer, discriminator_optimizer):
    noise = generate_noise(BATCH_SIZE)

    with tf.GradientTape() as disc_tape, tf.GradientTape() as gen_tape:
        generated_datas = generator(noise, training=False)
        fake_output = discriminator(generated_datas, training=True)
        real_output = discriminator(datas, training=True)
        disc_loss = discriminator_loss(real_output, fake_output)

    gradients_of_discriminator = disc_tape.gradient(disc_loss, discriminator.trainable_variables)
    discriminator_optimizer.apply_gradients(zip(gradients_of_discriminator, discriminator.trainable_variables))

    with tf.GradientTape() as gen_tape:
        generated_datas = generator(noise, training=True)
        fake_output = discriminator(generated_datas, training=False)
        gen_loss = generator_loss(fake_output)

    gradients_of_generator = gen_tape.gradient(gen_loss, generator.trainable_variables)
    generator_optimizer.apply_gradients(zip(gradients_of_generator, generator.trainable_variables))

    return gen_loss.numpy(), disc_loss.numpy()


def train_gan(generator, discriminator, df, batch_size, epochs):
    loss = {'generator': [], 'discriminator': []}
    best_epoch = 0
    best_gen_loss = float('inf')
```

```
    best_disc_loss = float('inf')

    early_stop_patience = epochs  # Early stopping patience
    early_stop_counter = 0  # Counter for epochs without improvement

    generator_optimizer = tf.keras.optimizers.Adam(learning_rate=GENERATOR_LEARNING_RATE)
    discriminator_optimizer = tf.keras.optimizers.Adam(learning_rate=DISCRIMINATOR_LEARNING_RATE)

    for epoch in range(epochs):
        start = time.time()
        epoch_gen_loss = []
        epoch_disc_loss = []

        df = df.sample(frac=1).reset_index(drop=True)  # Shuffle DataFrame

        for i in range(1, ceil(len(df) / batch_size) + 1):
            if i == ceil(len(df) / batch_size):
                train_data = df[:batch_size * i][
                    COLUMNS_RESPONSE + COLUMNS_VARIABLES + COLUMNS_INDICATOR].to_numpy()
            else:
                train_data = df[batch_size * (i - 1):batch_size * i][
                    COLUMNS_RESPONSE + COLUMNS_VARIABLES + COLUMNS_INDICATOR].to_numpy()

            loss_g, loss_d = train_step(train_data, generator, discriminator, generator_optimizer,
                                        discriminator_optimizer)
            epoch_gen_loss.append(loss_g)
            epoch_disc_loss.append(loss_d)

        avg_gen_loss = np.mean(epoch_gen_loss)
        avg_disc_loss = np.mean(epoch_disc_loss)
        loss['generator'].append(avg_gen_loss)
        loss['discriminator'].append(avg_disc_loss)

        if avg_gen_loss < best_gen_loss or avg_disc_loss < best_disc_loss:
            best_gen_loss = min(best_gen_loss, avg_gen_loss)
            best_disc_loss = min(best_disc_loss, avg_disc_loss)
            best_epoch = epoch
            generator.save(os.path.join(target_path, f'generator.keras'))
            discriminator.save(os.path.join(target_path, f'discriminator.keras'))
            early_stop_counter = 0
        else:
            early_stop_counter += 1

        print(
            f'epoch {epoch + 1:3}/{epochs} is {time.time() - start:.3f} sec || gen_loss = {avg_gen_loss:.4f},  disc_loss
= {avg_disc_loss:.4f}')

        if early_stop_counter >= early_stop_patience:  # Judge for early stopping
            print(f"Early stopping at epoch {epoch + 1}")
            break

        if epoch != 0 and epoch % 100 == 0:  # Temporary display of results
            print("Verification of GAN-synthesized data:")
            df_gan, df_gan_inv = generate_data(generator, preprocessor, 5)
            print(df_gan_inv)

    df_loss = pd.DataFrame(loss)
    plt.clf()
```

```
        plt.plot(df_loss)
        plt.title('Model Loss')
        plt.ylabel('Loss')
        plt.ylim(0, 2)
        plt.xlabel('Epoch')
        plt.legend(['generator', 'discriminator'], loc='upper right')
        LOSS_CSV_PATH = os.path.join(target_path, 'training_loss.csv')
        LOSS_GRAPH_PATH = os.path.join(target_path, 'training_loss.png')
        df_loss.to_csv(LOSS_CSV_PATH)
        plt.savefig(LOSS_GRAPH_PATH)


    # Generating synthetic data
    def generate_data(generator, preprocessor, num_samples):
        noise = generate_noise(num_samples)

        pred = generator.predict(noise)
        df_gan = pd.DataFrame(data=pred, columns=COLUMNS_RESPONSE + COLUMNS_VARIABLES +
COLUMNS_INDICATOR)

        # Add tentative index
        df_gan.insert(0, COLUMNS_INDEX[0], range(1, 1 + num_samples))

        # Inverse transform numeric variables in the DataFrame
        numeric_columns = [col for col in COLUMNS_RESPONSE + COLUMNS_VARIABLES if col not in
COLUMNS_CAT]
        df_num =
pd.DataFrame(preprocessor.named_transformers_['num'].inverse_transform(df_gan[numeric_columns]),
                              columns=numeric_columns)

        # Clip numeric variables for reasonable distribution
        df_num = df_num.clip(lower=0)
        # df_num[COLUMNS_RESPONSE] = df_num[COLUMNS_RESPONSE].clip(lower=15, upper=30)

        # Limit the values of categorical and indicator variables to 0 or 1
        df_gan[COLUMNS_CAT + COLUMNS_INDICATOR] = np.where(df_gan[COLUMNS_CAT +
COLUMNS_INDICATOR] < 0.5, 0, 1).astype(
            int)
        # df_gan[COLUMNS_CAT + COLUMNS_INDICATOR] = df_gan[COLUMNS_CAT +
COLUMNS_INDICATOR].astype(int)
        # print(df_gan.dtypes)

        df_gan_inv = pd.concat(
            [df_gan[COLUMNS_INDEX].reset_index(drop=True), df_num.reset_index(drop=True),
             df_gan[COLUMNS_CAT + COLUMNS_INDICATOR].reset_index(drop=True)], axis=1)

        # # Limit the values of categorical and indicator variables to 0 or 1
        # for col in COLUMNS_CAT + COLUMNS_INDICATOR:
        #     df_gan_inv[col] = df_gan_inv[col].apply(lambda x: 0 if x < 0.5 else 1)

        # print(df_gan_inv.head())
        return df_gan, df_gan_inv


    # Function to instantiate Generator and Discriminator
    def initialize_gan_models():
        generator = Generator().build()
        discriminator = Discriminator().build()
```

```
        return generator, discriminator


    '''
    Iteration of data augmentation by random_seed, size of synthesized data, and the methods (SMOGN, GAN, and
CTGAN)
    '''
    random_seed = INIT_RANDOM_SEED
    for epochs in LIST_GAN_EPOCHS:
        for num_seed in range(0, 20):
            random_seed = INIT_RANDOM_SEED + num_seed

            target_path = os.path.join(BASE_DIR, 'EPOCHS{}SEED{}'.format(epochs, random_seed))
            if not os.path.exists(target_path):
                os.mkdir(target_path)

            # Split and save the training and validation data
            train_df, test_df = train_test_split(df, test_size=TEST_SIZE, random_state=random_seed)
            train_df_norm, test_df_norm, preprocessor = normalize_train_test_data(train_df, test_df, COLUMNS_INDEX,
                                                                                  COLUMNS_RESPONSE, COLUMNS_VARIABLES,
                                                                                  COLUMNS_INDICATOR, COLUMNS_CAT)

            train_df.to_csv(os.path.join(target_path, f'train.csv'), index=False, encoding='shift-jis')
            train_df_norm.to_csv(os.path.join(target_path, f'train_norm.csv'), index=False, encoding='shift-jis')
            test_df.to_csv(os.path.join(target_path, f'test.csv'), index=False, encoding='shift-jis')
            test_df_norm.to_csv(os.path.join(target_path, f'test_norm.csv'), index=False, encoding='shift-jis')

            # Display standardization results
            print("Training data (original):")
            print(train_df.head())
            print("Training data (normalized):")
            print(train_df_norm.head())

            print("Test data (original):")
            print(test_df.head())
            print("Test data (normalized):")
            print(test_df_norm.head())
            time.sleep(2)

            train_df = pd.read_csv(os.path.join(target_path, f'train.csv'), encoding='shift-jis')
            train_df_norm = pd.read_csv(os.path.join(target_path, f'train_norm.csv'), encoding='shift-jis')

            # Initializing meta data
            metadata = SingleTableMetadata()
            metadata.detect_from_dataframe(train_df_norm)
            metadata.update_column(column_name='MMSE', sdtype='numerical')
            # metadata.update_column(column_name='PISA', sdtype='numerical')
            # metadata.update_column(column_name='PESA', sdtype='numerical')
            metadata.update_column(column_name='Sex', sdtype='categorical')
            # metadata.update_column(column_name='TP_isNA', sdtype='categorical')
            # metadata.update_column(column_name='HbA1c_isNA', sdtype='categorical')
            metadata_file_path = os.path.join(target_path, 'metadata.json')
            if os.path.exists(metadata_file_path):
                os.remove(metadata_file_path)
            metadata.save_to_json(metadata_file_path)
            print(metadata)

            '''
```

```
    SMOTE for Regression (SMOGN)
    '''

    print("SMOGN:")
    train_df = pd.read_csv(os.path.join(target_path, f'train.csv'), encoding='shift-jis')
    train_df_norm = pd.read_csv(os.path.join(target_path, f'train_norm.csv'), encoding='shift-jis')
    # train_df_norm_smogn = train_df_norm[COLUMNS_RESPONSE + COLUMNS_VARIABLES +
COLUMNS_INDICATOR]

    def dynamic_relevance_function(y):
        # Calculate quartiles
        q0 = y.min()
        q1 = np.percentile(y, 25)
        q2 = np.percentile(y, 50)
        q3 = np.percentile(y, 75)
        q4 = y.max()
        if q0 == q1:
            q1 += 1e-6
        if q1 >= q2:
            q2 = q1 + 1e-6
        if q2 >= q3:
            q3 = q2 + 1e-6
        if q3 >= q4:
            q4 = q3 + 1e-6

        relevance = [
            [q0, 0, 0],
            [q1, 0.5, 0],
            [q2, 1, 0],
            [q3, 0.5, 0],
            [q4, 0, 0]
        ]
        return relevance


    df_smogn = smogn.smoter(
        data=train_df_norm[COLUMNS_RESPONSE + COLUMNS_VARIABLES + COLUMNS_INDICATOR],
        y=COLUMNS_RESPONSE[0],  # Column name of the target variable
        samp_method='balance',
        # samp_method = 'extreme',
        # rel_coef = 1.2,
        # rel_thres = 0.5,
        rel_method='manual',
        rel_ctrl_pts_rg=dynamic_relevance_function(train_df_norm['MMSE'])
    )
    df_smogn.reset_index(drop=True, inplace=True)

    # Reverse transformation and clipping of numeric variables in the dataframe before standardization
    numeric_columns = [col for col in COLUMNS_RESPONSE + COLUMNS_VARIABLES if col not in
COLUMNS_CAT]
    df_num =
pd.DataFrame(preprocessor.named_transformers_['num'].inverse_transform(df_smogn[numeric_columns]),
                    columns=numeric_columns)
    df_num = df_num.clip(lower=0)

    # Add index
    df_num.insert(0, COLUMNS_INDEX[0], range(1, len(df_num) + 1))
```

```
        # Restrict values of categorical variables and indicator variables to 0 or 1
        df_smogn[COLUMNS_CAT + COLUMNS_INDICATOR] = np.where(df_smogn[COLUMNS_CAT +
COLUMNS_INDICATOR] < 0.5, 0, 1).astype(
            int)

        df_smogn_inv = pd.concat(
            [df_num.reset_index(drop=True),
             df_smogn[COLUMNS_CAT + COLUMNS_INDICATOR].reset_index(drop=True)], axis=1)

        # Saving SMOGN-synthetic data
        # df_smogn.to_csv(os.path.join(target_path, f'smogn_synthetic_data_norm.csv'), index=False)
        df_smogn_inv.to_csv(os.path.join(target_path, f'smogn_synthetic_data.csv'), index=False)
        print("Dataframe (first 5):")
        print(df_smogn_inv.head())


        quality_report = evaluate_quality(
            real_data=train_df,
            synthetic_data=df_smogn_inv,
            metadata=metadata)

        with open(os.path.join(target_path, f'smogn_synthetic_data_quality_report.txt'), 'w') as f:
            print(quality_report.get_info(), file=f)
            print(file=f)
            print(quality_report.get_score(), file=f)
            print(quality_report.get_properties(), file=f)
            print(file=f)
            print(quality_report.get_details(property_name='Column Shapes'), file=f)
            # print(quality_report.get_details(property_name='Column Pair Trends'), file=f)
            f.write(quality_report.get_details(property_name='Column Pair Trends').to_string())

        # Visualization of the results
        if not df_smogn_inv[COLUMNS_RESPONSE[0]].empty and not
train_df[COLUMNS_RESPONSE[0]].empty:
            fig = get_column_plot(real_data=train_df, synthetic_data=df_smogn_inv, column_name='MMSE',
                                  metadata=metadata)
            fig.write_html(os.path.join(target_path, f'smogn_synthetic_data.html'))

        '''
        Generating synthetic data using Normal GAN
        '''
        print("Normal GAN - " + 'EPOCHS{}, SEED{}'.format(epochs, random_seed))

        # GAN training
        generator, discriminator = initialize_gan_models()
        train_gan(generator, discriminator, train_df_norm, BATCH_SIZE, epochs)

        for gan_size in LIST_GAN_SIZE:
            # Generate synthetic data
            df_gan, df_gan_inv = generate_data(generator, preprocessor, gan_size)

            # df_gan.to_csv(os.path.join(target_path, f'gan_synthetic_data_{i}_norm.csv'), index=False,
            #               encoding='shift-jis')
            df_gan_inv.to_csv(os.path.join(target_path, f'gan_synthetic_data_{gan_size}.csv'), index=False,
                              encoding='shift-jis')

            # Visualization of the results
```

```
            if not df_gan_inv[COLUMNS_RESPONSE[0]].empty and not
train_df[COLUMNS_RESPONSE[0]].empty:
                fig = get_column_plot(real_data=train_df, synthetic_data=df_gan_inv, column_name='MMSE',
                                      metadata=metadata)
                fig.write_html(os.path.join(target_path, f'gan_synthetic_data_{gan_size}.html'))

            quality_report = evaluate_quality(
                real_data=train_df,
                synthetic_data=df_gan_inv,
                metadata=metadata)

            with open(os.path.join(target_path, f'gan_synthetic_data_{gan_size}_quality_report.txt'), 'w') as f:
                print(quality_report.get_info(), file=f)
                print(file=f)
                print(quality_report.get_score(), file=f)
                print(quality_report.get_properties(), file=f)
                print(file=f)
                print(quality_report.get_details(property_name='Column Shapes'), file=f)
                # print(quality_report.get_details(property_name='Column Pair Trends'), file=f)
                f.write(quality_report.get_details(property_name='Column Pair Trends').to_string())

        '''
        # Generating synthetic data using CTGAN
        '''
        print("CTGAN - " + 'EPOCHS{}, SEED{}'.format(epochs, random_seed))

        ctgan = CTGANSynthesizer(metadata=metadata, epochs=epochs, verbose=True)

        # # Constraints to generate output data
        # my_constraint = {
        #     'constraint_class': 'ScalarInequality',
        #     'constraint_parameters': {
        #         'column_name': 'MMSE',
        #         'relation': '>=',
        #         'value': 15
        #     }
        # }
        # ctgan.add_constraints(constraints=[my_constraint])
        # ctgan.get_constraints()

        # Fit the CTGAN model
        ctgan.fit(train_df)

        for gan_size in LIST_GAN_SIZE:
            df_ctgan = ctgan.sample(gan_size)

            df_ctgan.to_csv(os.path.join(target_path, f'ctgan_synthetic_data_{gan_size}.csv'), index=False,
                            encoding='shift-jis')

            # Visualization of the results
            if not df_ctgan[COLUMNS_RESPONSE[0]].empty and not train_df[COLUMNS_RESPONSE[0]].empty:
                fig = get_column_plot(real_data=train_df, synthetic_data=df_ctgan, column_name='MMSE',
                                      metadata=metadata)
                fig.write_html(os.path.join(target_path, f'gan_synthetic_data_{gan_size}.html'))

            quality_report = evaluate_quality(
                real_data=train_df,
                synthetic_data=df_ctgan,
```

```
            metadata=metadata)

        with open(os.path.join(target_path, f'ctgan_synthetic_data_{gan_size}_quality_report.txt'), 'w') as f:
            print(quality_report.get_info(), file=f)
            print(file=f)
            print(quality_report.get_score(), file=f)
            print(quality_report.get_properties(), file=f)
            print(file=f)
            print(quality_report.get_details(property_name='Column Shapes'), file=f)
            f.write(quality_report.get_details(property_name='Column Pair Trends').to_string())
```

List S2. Repeated Hold-Out Validation Using LR, RF, and DNN

Introduction:

This script performs repeated hold-out validation using three machine learning models: Linear Regression (LR), Random Forest (RF), and Deep Neural Network (DNN), to create SMOGN-, GAN-, and CTGAN-synthesized models. The key variables used include LIST_EPOCHS, LIST_TRAINING_DATA, and Iter_SEED for iterating over different configurations of the training process during the repeated hold-out validations.

Variables and Columns:

ROBUSTNESS_TEST: Set to False for this analysis.

COLUMNS_INDEX: ['n']
COLUMNS_RESPONSE: ['MMSE']
COLUMNS_CAT: ['Sex'] (Categorical variable)
LIST_COLUMNS_VARIABLES: Two lists of variable columns to be used in the validation.

Data Preparation

(1) Loading Labeled Dataset:
The labeled dataset is loaded from the specified CSV file.
Invalid data (rows with missing MMSE values) is removed.
Missing values are filled with the mean of each column.
(2) Scaling Data:
The dataset is scaled using StandardScaler from the sklearn library to normalize the feature values.

Repeated Hold-Out Validation

INIT_RANDOM_SEED: 100
NUM_ITER: 10
LIST_EPOCHS: [300, 1000, 3000, 5000]
LIST_TRAINING_DATA: ['gan_synthetic_data_100.csv', 'gan_synthetic_data_500.csv']

For each combination of epochs and training data, the following steps are repeated for NUM_ITER iterations:

(1) Set Random Seed:
A random seed is set to ensure reproducibility.
(2) Load Synthesized Datasets:
The training and test datasets are loaded from CSV files. The training dataset may include real data, synthetic data, or a combination of both.
(3) External Validation Dataset:
An external validation dataset is loaded to evaluate the model's performance on unseen data.
(4)Model Training and Evaluation:
Linear Regression (LR):
A linear regression model is trained and evaluated.
Model coefficients and permutation importance scores are calculated.

Random Forest (RF):
A random forest regressor is trained and evaluated.
Model feature importances and permutation importance scores are calculated.
Deep Neural Network (DNN):
A DNN is constructed and trained with specified parameters.
Training history, mean absolute error (MAE), and other metrics are recorded.
Permutation importance scores are calculated.

Code:

```
'''
Analysis of Blood Tests and Periodontal Examination Data
Comparison of Machine Learning Results
(Frontiers in Neurology)
May, 2024
'''

from __future__ import absolute_import, division, print_function, unicode_literals

import os
from pprint import pprint
from glob import glob

import matplotlib.pyplot as plt
import numpy as np
import pandas as pd
import sklearn
import tensorflow as tf
from scipy.stats import pearsonr, spearmanr, ttest_ind
from sklearn import preprocessing
from sklearn.utils import shuffle
from sklearn.metrics import mean_squared_error
from sklearn.metrics import mean_absolute_error
from sklearn.linear_model import LinearRegression
from sklearn.linear_model import LassoCV, RidgeCV
from sklearn.linear_model import Lasso
from sklearn.svm import SVR
from sklearn.ensemble import RandomForestRegressor
from sklearn.model_selection import GridSearchCV
from sklearn.model_selection import cross_val_score
from statsmodels.stats.outliers_influence import variance_inflation_factor
import statsmodels.api as sm

pd.set_option('display.max_rows', None)

'''
Running Parameters
'''
# Value of Age will be set to 0 in this robustness test
ROBUSTNESS_TEST = False

# # Input file
RealData_filename = 'digitalhealth_data2023_0519_ZeroToNa.csv'
ExternalValidation_filename = 'digitalhealth_SK20220520_KoriyamaHokenjo.csv'

DATA_DIR = 'data'
os.makedirs(DATA_DIR, exist_ok=True)

BASE_DIRS = [os.path.join(DATA_DIR, 'RepeatedHoldOut_CTGAN_0607Ver3_{}'.format(
```

```
    RealData_filename.rsplit('.', 1)[0])),
            os.path.join(DATA_DIR,
                         'RepeatedHoldOut_CTGAN_0607Ver3a_without_PeriodontalExam_{}'.format(
                             RealData_filename.rsplit('.', 1)[0]))]
BASE_DIR = BASE_DIRS[0]

COLUMNS_INDEX = ['n']
COLUMNS_RESPONSE = ['MMSE']
COLUMNS_CAT = ['Sex']  # Categorical variables

LIST_COLUMNS_VARIABLES = [
    ['Age',
     'WBC', 'Hb', 'Plt', 'AST', 'ALT',
     'LDH',
     # 'GTT', 'CK',
     'T-Cho',
     'HDL', 'LDL',
     'TG', 'BUN', 'Cr', 'UA',
     'TP',
     # 'hsCRP', 'TropT', 'NTproBNP',
     'HbA1c',
     # 'dROM', 'BAP',
     'Height', 'Weight',
     # 'BMI',
     'sBP', 'dBP',
     'HR',

     'RemainingTooth',
     'AvePD',
     'AveCAL',
     # 'BOP',
     'PISA',
     'PESA'
    ] + COLUMNS_CAT,
    ['Age', 'WBC', 'Hb', 'Plt', 'AST', 'ALT',
     'LDH',
     # 'GTT', 'CK',
     'T-Cho',
     'HDL', 'LDL',
     'TG', 'BUN', 'Cr', 'UA',
     'TP',
     # 'hsCRP', 'TropT', 'NTproBNP',
     'HbA1c',
     # 'dROM', 'BAP',
     'Height', 'Weight',
     # 'BMI',
     'sBP', 'dBP',
     'HR'
     ] + COLUMNS_CAT
]

tf.keras.backend.clear_session()
print('Tensorflow ' + tf.__version__)
print("Eager mode: ", tf.executing_eagerly())
print("GPU is", "available" if tf.config.list_physical_devices('GPU') else "NOT AVAILABLE")
print('\n')

for loop_index in range(0, 1):
```

```
COLUMNS_VARIABLES = LIST_COLUMNS_VARIABLES[loop_index]
BASE_DIR = BASE_DIRS[loop_index]

'''
Labeled Training dataset
'''
print('Labeled dataset: ' + os.path.join(DATA_DIR, RealData_filename))
labeled_dataset0 = pd.read_csv(os.path.join(DATA_DIR, RealData_filename), sep=",", encoding="shift-jis")

pprint(labeled_dataset0)
print("Dataset loading - complete\n")

# Remove invalid data
print("Dataset after removal of invalid data")
labeled_dataset_dropna = labeled_dataset0.dropna(subset=['MMSE'])
print(labeled_dataset_dropna)
print("Columns of NA (after the removal of invalid data)")
pprint(labeled_dataset_dropna.isna().sum())

labeled_dataset = labeled_dataset_dropna
pprint(labeled_dataset.isna().sum())
pprint(labeled_dataset)
print("Dataset for processing")


def training_stats(dataset, filename='stats', outputDir=BASE_DIR):
    training_stats = dataset.describe().transpose()
    training_stats.to_csv(os.path.join(outputDir, filename))
    print('Statistics:')
    pprint(training_stats.loc[:, :'std'])
    print('\n')
    return training_stats


labeled_dataset_stats = training_stats(labeled_dataset, 'training_stats_' + RealData_filename, BASE_DIR)

labeled_dataset_fillna = labeled_dataset.fillna(labeled_dataset_stats['mean'])


def my_scaler(X, df_columns):
    scaler = preprocessing.StandardScaler()
    x_sc = scaler.fit_transform(X)
    x_sc = pd.DataFrame(x_sc, columns=df_columns)
    return x_sc


X = labeled_dataset_fillna[COLUMNS_VARIABLES]
X_sc = my_scaler(X, COLUMNS_VARIABLES)
y = labeled_dataset_fillna[COLUMNS_RESPONSE].values.ravel()

'''
Basic Analysis using the Original Data before Splitting
'''
# Significance Testing
labeled_dataset_group1 = labeled_dataset[labeled_dataset['MMSE'] >= 28].reset_index(drop=True)
labeled_dataset_group2 = labeled_dataset[labeled_dataset['MMSE'] < 28].reset_index(drop=True)

training_stats(labeled_dataset_group1, 'training_stats1.csv', BASE_DIR)
```

```
training_stats(labeled_dataset_group2, 'training_stats2.csv', BASE_DIR)

results_df = pd.DataFrame(columns=['Variable', 't_statistic', 'p_value'])

filtered_columns = [column for column in COLUMNS_VARIABLES if column not in COLUMNS_CAT]
for column in filtered_columns:
    data1 = labeled_dataset_group1[column].dropna()
    data2 = labeled_dataset_group2[column].dropna()
    t_stat, p_value = ttest_ind(data1, data2, equal_var=False, alternative='greater')

    new_row = pd.DataFrame({'Variable': [column], 't_statistic': [t_stat], 'p_value': [p_value]})
    # Check if new_row has all NA columns
    if not new_row.isnull().all(axis=1).all():
        results_df = pd.concat([results_df, new_row], ignore_index=True)

results_df.to_csv(os.path.join(BASE_DIR, 't_test_results.csv'), index=False)


# Correlation Analysis
def calculate_p_values(df, method='pearson'):
    df = df.dropna()._get_numeric_data()
    cols = df.columns
    p_values = pd.DataFrame(columns=cols, index=cols)
    for r in cols:
        for c in cols:
            if r == c:
                p_values.loc[r, c] = np.nan
            else:
                # Check if both columns have at least 2 non-null values
                if df[r].notnull().sum() < 2 or df[c].notnull().sum() < 2:
                    p_values.loc[r, c] = np.nan
                else:
                    if method == 'pearson':
                        _, p = pearsonr(df[r], df[c])
                    else:
                        _, p = spearmanr(df[r], df[c])
                    p_values.loc[r, c] = p
    return p_values.astype(float)  # Ensure the DataFrame is of float type


# Drop columns with fewer than 2 non-null values
labeled_dataset_fillna = labeled_dataset.dropna(thresh=2, axis=1).fillna(labeled_dataset_stats['mean'])

# Extract numeric columns for correlation analysis
numeric_columns = labeled_dataset_fillna.select_dtypes(include=[np.number]).columns

labeled_dataset_fillna[numeric_columns].corr(method='pearson').to_csv(
    os.path.join(BASE_DIR, 'CorrelationMatrix_Pearson.csv'), float_format='%.2f')
calculate_p_values(labeled_dataset_fillna[numeric_columns], method='pearson').to_csv(
    os.path.join(BASE_DIR, 'CorrelationMatrix_Pearson_p_values.csv'), float_format='%.3f')
labeled_dataset_fillna[numeric_columns].corr(method='spearman').to_csv(
    os.path.join(BASE_DIR, 'CorrelationMatrix_Spearman.csv'), float_format='%.2f')
calculate_p_values(labeled_dataset_fillna[numeric_columns], method='spearman').to_csv(
    os.path.join(BASE_DIR, 'CorrelationMatrix_Spearman_p_values.csv'), float_format='%.3f')

def permutation_importance(model, X_val, y_val, metric=mean_absolute_error, n_iter=30):
    baseline = metric(y_val, model.predict(X_val))
    scores = np.zeros(X_val.shape[1])
```

```
    for i in range(X_val.shape[1]):
        score_changes = []
        for _ in range(n_iter):
            X_val_permuted = X_val.copy()
            if isinstance(X_val, pd.DataFrame):
                X_val_permuted.iloc[:, i] = shuffle(X_val_permuted.iloc[:, i])
            else:
                X_val_permuted[:, i] = shuffle(X_val_permuted[:, i])
            new_score = metric(y_val, model.predict(X_val_permuted))
            score_changes.append(new_score - baseline)
        scores[i] = np.mean(score_changes)
    return scores

'''
Feature analysis
'''
# (1) Checking VIF
vif_data = pd.DataFrame()
vif_data["feature"] = X_sc.columns
vif_data["VIF"] = [variance_inflation_factor(X_sc.values, i) for i in range(X_sc.shape[1])]
print(vif_data)

# (2) Lasso and Ridge analysis to find important variables
lasso = LassoCV(alphas=[0.1, 0.5, 1.0, 2.0, 3.0, 10.0], cv=10)
lasso.fit(X, y)
lasso_scores = cross_val_score(lasso, X, y, cv=10, scoring='neg_mean_absolute_error')
mean_mae_lasso = -lasso_scores.mean()
std_mae_lasso = lasso_scores.std()
print(f'Mean MAE (Lasso): {mean_mae_lasso:.2f} ± {std_mae_lasso:.2f}')
lasso_coefs = pd.Series(lasso.coef_, index=COLUMNS_VARIABLES)

ridge = RidgeCV(alphas=[0.1, 1.0, 10.0])
ridge.fit(X, y)
ridge_scores = cross_val_score(ridge, X, y, cv=10, scoring='neg_mean_absolute_error')
mean_mae_ridge = -ridge_scores.mean()
std_mae_ridge = ridge_scores.std()
print(f'Mean MAE (Ridge): {mean_mae_ridge:.2f} ± {std_mae_ridge:.2f}')
ridge_coefs = pd.Series(ridge.coef_, index=COLUMNS_VARIABLES)

print("\nImportant Variables in both Lasso and Ridge Regression:")
important_variables = lasso_coefs[lasso_coefs != 0].index
important_variables_ridge = ridge_coefs.loc[important_variables]
print(important_variables_ridge)
results_df = pd.DataFrame({
    'Ridge Coefficients': ridge_coefs,
    'Lasso Coefficients': lasso_coefs
})
results_df.sort_values(by='Ridge Coefficients', ascending=False)
print(results_df)

'''
DNN model Analysis
'''
def compileModel(model):
    opt = tf.keras.optimizers.Adam(learning_rate=0.001, beta_1=0.9, beta_2=0.999)

    model.compile(loss='mse',
              optimizer=opt,
```

```
                  metrics=['mae'])

        return model


    def getModel(target_x, Num_Classes=2, loadModel=False, modelFilePath=""):
        model = tf.keras.Sequential()
        model.add(tf.keras.Input(target_x.shape[1:], name='feature'))
        model.add(tf.keras.layers.GaussianNoise(0.01))

        for units in [256, 128, 64]:
            model.add(tf.keras.layers.BatchNormalization())
            model.add(
                tf.keras.layers.Dense(units, kernel_regularizer=tf.keras.regularizers.l2(0.001), activation='selu'))
            model.add(tf.keras.layers.Dropout(0.1))

        model.add(tf.keras.layers.BatchNormalization())
        model.add(tf.keras.layers.Dense(1))

        model = compileModel(model)

        if loadModel:
            model.load_weights(modelFilePath)

        return model


    # Acquisition of the model
    model = getModel(labeled_dataset_fillna.values, 2)
    print(model.summary())
    print('')


    # train the model
    def trainModel(model, x_train, y_train, batch_size=8, epochs=300, patience=20, best_only_mode=True):
        # es_cb = tf.keras.callbacks.EarlyStopping(monitor='val_loss', patience=patience, verbose=1, mode='auto',
restore_best_weights=True)
        lr_cb = tf.keras.callbacks.ReduceLROnPlateau(monitor='val_loss', factor=0.9, patience=patience // 2, verbose=1,
                                                     mode='auto', min_lr=1e-5)
        chkpt = os.path.join(BASE_DIR,
                             'best_model.keras')
        cp_cb = tf.keras.callbacks.ModelCheckpoint(filepath=chkpt, monitor='val_loss', verbose=1,
                                                   save_best_only=best_only_mode,
                                                   mode='auto', save_freq='epoch')

        # Save the training history
        history = model.fit(x_train, y_train,
                            batch_size=batch_size,
                            epochs=epochs,
                            verbose=1,
                            validation_split=0.2,  # Use 20% of the training data as validation data
                            # callbacks=[es_cb, lr_cb, cp_cb],
                            callbacks=[lr_cb, cp_cb],
                            shuffle=True)

        return model, history
```

```
'''
Iterations of Repeated Hold-Out Validation
'''
INIT_RANDOM_SEED = 100
NUM_ITER = 10
LIST_EPOCHS = [300, 1000, 3000, 5000]
LIST_TRAINING_DATA = ['train.csv', 'smogn_synthetic_data.csv', 'gan_synthetic_data_100.csv',
                 'gan_synthetic_data_200.csv', 'gan_synthetic_data_500.csv', 'ctgan_synthetic_data_100.csv',
'ctgan_synthetic_data_200.csv',
                 'ctgan_synthetic_data_500.csv']

for i in LIST_EPOCHS:
    for j in LIST_TRAINING_DATA:
        for Iter_SEED in range(INIT_RANDOM_SEED, INIT_RANDOM_SEED + NUM_ITER):
            seed = Iter_SEED
            np.random.seed(seed)

            '''
            Loading Synthesized Datasets
            '''
            # Setting the basic path
            filepath2_test = 'test.csv'
            filepath2_train_real = 'train.csv'
            filepath2_train_synthetic = j
            filepath2_base = BASE_DIR + '/EPOCHS' + str(i) + 'SEED' + str(seed)

            # Loading extended datasets
            train_real_data = \
                pd.read_csv(os.path.join(filepath2_base, filepath2_train_real), sep=",", encoding="shift-jis")[
                    COLUMNS_RESPONSE + COLUMNS_VARIABLES]

            train_synthetic_data = \
                pd.read_csv(os.path.join(filepath2_base, filepath2_train_synthetic), sep=",", encoding="shift-jis")[
                    COLUMNS_RESPONSE + COLUMNS_VARIABLES]
            if j == 'train.csv':
                train_dataset = train_real_data
            else:
                train_dataset = pd.concat([train_real_data, train_synthetic_data], ignore_index=True)
            test_dataset = pd.read_csv(os.path.join(filepath2_base, filepath2_test), sep=",", encoding="shift-jis")[
                COLUMNS_RESPONSE + COLUMNS_VARIABLES]

            '''
            External Validation Test Dataset
            '''
            print('External Validation Test dataset: ' + os.path.join(DATA_DIR, ExternalValidation_filename))
            ex_test_dataset0 = pd.read_csv(os.path.join(DATA_DIR, ExternalValidation_filename), sep=",",
                                           encoding="shift-jis")

            # Correct and specify the type of the categorical variable 'Sex'
            ex_test_dataset0['Sex'] = ex_test_dataset0['Sex'].apply(
                lambda x: 0 if x == 'Male' else (1 if x == 'Female' else x)
            ).astype('category')

            pprint(ex_test_dataset0)
            print("Dataset loading complete")
            print('\n')

            # Values of categorical columns
```

```
categorical_columns = ex_test_dataset0.select_dtypes(['category']).columns

# Add items that do not exist in ex_test_dataset and fill missing values with the mean or mode
for column in train_dataset.columns:
    if column not in ex_test_dataset0.columns:
        if column in categorical_columns:
            ex_test_dataset0[column] = train_dataset[column].mode()[0]
            ex_test_dataset0[column] = ex_test_dataset0[column].astype('category')
            ex_test_dataset0[column] = ex_test_dataset0[column].cat.add_categories(
                [train_dataset[column].mode()[0]])
        else:
            ex_test_dataset0[column] = train_dataset[column].mean()
    else:
        if column in categorical_columns:
            ex_test_dataset0[column] = ex_test_dataset0[column].astype('category')
            ex_test_dataset0[column].fillna(train_dataset[column].mode()[0], inplace=True)
        else:
            ex_test_dataset0[column].fillna(train_dataset[column].mean(), inplace=True)

ex_test_dataset = ex_test_dataset0[COLUMNS_RESPONSE + COLUMNS_VARIABLES]

'''
For robustness tests
'''
if ROBUSTNESS_TEST == True:
    Column_AnomalousInput = 'Age'
    Value_AnomalousInput = 0
    test_dataset.loc[:, Column_AnomalousInput] = Value_AnomalousInput
    ex_test_dataset.loc[:, Column_AnomalousInput] = Value_AnomalousInput

'''
Saving test dataset
'''
train_dataset.to_csv(os.path.join(filepath2_base, 'train_dataset_filled.csv'), index=False)
ex_test_dataset.to_csv(os.path.join(filepath2_base, 'ex_test_dataset_filled.csv'), index=False)

print("train_dataset:")
print(train_dataset.head())
print("ex_test_dataset:")
print(ex_test_dataset.head())

'''
Loading training and test datasets
'''
print("Loading training and test datasets:")
print(f"{filepath2_base} - Training data: {filepath2_train_synthetic} Test data: {filepath2_test}")

# Save statistical information
training_stats = train_dataset.describe()[COLUMNS_RESPONSE + COLUMNS_VARIABLES].transpose()
stats_file_path = os.path.join(filepath2_base, f'training_stats.csv')
training_stats.to_csv(stats_file_path)

print('\n')


'''
Repeated Hold-Out validation 1 - Linear Regression using Synthesized data (SMOGN, GAN, and CTGAN)
'''
```

```
    Num_Classes = 2
    N_Iterations = 1  # For k-fold CV (Set to 1 for Hold-Out validation)

    # Model selection
    model_name = 'LR'
    model = LinearRegression()

    # Iterations of Hold-Out Validation
    for k in range(N_Iterations):
        print(model_name + " - Hold-Out", str(k), " -------------------------------------------")

        result_dfs = []  # List to store results
        result_coefficient_dfs = []  # List to store coefficients
        model_coefficients_dfs = []

        x_train = train_dataset[COLUMNS_VARIABLES]
        scaler = sklearn.preprocessing.StandardScaler()
        x_train_norm_values = scaler.fit_transform(x_train)
        y_train = train_dataset[COLUMNS_RESPONSE]
        x_test = test_dataset[COLUMNS_VARIABLES]
        x_test_norm_values = scaler.transform(x_test)
        y_test = test_dataset[COLUMNS_RESPONSE]

        # Training
        model = LinearRegression()
        model.fit(x_train_norm_values, y_train)

        y_pred = model.predict(x_test_norm_values)
        # Add DataFrame to the list
        df = pd.merge(pd.DataFrame(y_test.values), pd.DataFrame(y_pred), left_index=True, right_index=True)
        df = pd.merge(pd.DataFrame(df), pd.DataFrame(x_test).reset_index(drop=True), left_index=True,
                right_index=True)
        result_dfs.append(df)

        # Save results to a CSV file
        mae = np.mean(np.abs(df.iloc[:, 1] - df.iloc[:, 0]))
        rmse = np.sqrt(mean_squared_error(df.iloc[:, 0], df.iloc[:, 1]))
        cor = df.corr().iloc[0, 1]

        # Get model intercept and coefficients, and add them to the list
        df.to_csv(
            os.path.join(filepath2_base,
                    f'{filepath2_train_synthetic} - {model_name} -
Round{k}_MAE{mae:.3f}_RMSE{rmse:.3f}_COR{cor:.3f}.csv'))

        # Get model intercept and coefficients, and add them to the list
        model_coefficients_dfs.append(pd.DataFrame([np.append(model.intercept_, model.coef_)],
                                    columns=['Intercept'] + COLUMNS_VARIABLES))
        model_coefficients_dfs[0].to_csv(
            os.path.join(filepath2_base, f'{filepath2_train_synthetic} - {model_name} - Coefficients.csv'))

        # Permutation Importance
        scores = permutation_importance(model, x_test_norm_values, y_test.values)
        result_coefficient_dfs.append(pd.DataFrame([scores], columns=x_test.columns))

        # For external tests
        x_ex_test = ex_test_dataset[COLUMNS_VARIABLES]
        x_ex_test_norm_values = scaler.transform(x_ex_test)
```

```
        y_ex_test = ex_test_dataset[COLUMNS_RESPONSE]

        y_ex_pred = model.predict(x_ex_test_norm_values)

        df_ex = pd.merge(pd.DataFrame(y_ex_test.values), pd.DataFrame(y_ex_pred), left_index=True,
                    right_index=True)
        df_ex = pd.merge(pd.DataFrame(df_ex), pd.DataFrame(x_ex_test).reset_index(drop=True),
                    left_index=True,
                    right_index=True)

        mae = np.mean(np.abs(df_ex.iloc[:, 1] - df_ex.iloc[:, 0]))
        rmse = np.sqrt(mean_squared_error(df_ex.iloc[:, 0], df_ex.iloc[:, 1]))
        cor = df_ex.corr().iloc[0, 1]

        output_file = f'{filepath2_train_synthetic} - {model_name} - External Test -
Round{k}_MAE{mae:.3f}_RMSE{rmse:.3f}_COR{cor:.3f}.csv'
        print(output_file)
        df_ex.to_csv(
          os.path.join(filepath2_base,
                    f'{filepath2_train_synthetic} - {model_name} - External Test -
Round{k}_MAE{mae:.3f}_RMSE{rmse:.3f}_COR{cor:.3f}.csv'))

      # Combine DataFrames in the list
      df_correlation = pd.concat(result_dfs, ignore_index=True)
      result_df_coefficients = pd.concat(result_coefficient_dfs, ignore_index=True)

      # Calculate MAE, MSE, and COR for the combined results DataFrame
      total_mae = np.mean(np.abs(df_correlation.iloc[:, 1] - df_correlation.iloc[:, 0]))
      total_rmse = np.sqrt(mean_squared_error(df_correlation.iloc[:, 0], df_correlation.iloc[:, 1]))
      total_cor = df_correlation.corr().iloc[0, 1]

      output_file = f'{filepath2_train_synthetic} - {model_name} - Repeated Hold-Out Validation - {Iter_SEED} -
MAE{total_mae:.3f}_RMSE{total_rmse:.3f}_COR{total_cor:.3f}'
      print(output_file)
      df_correlation.to_csv(os.path.join(filepath2_base, f'{output_file}.csv'))

      # Combine coefficients DataFrames
      model_coefficients_df = pd.concat(model_coefficients_dfs, ignore_index=True)
      model_coefficients_df.to_csv(
        os.path.join(filepath2_base, f'{filepath2_train_synthetic} - {model_name} - Coefficients.csv'))

      result_df_coefficients.to_csv(
        os.path.join(filepath2_base,
                  f'{filepath2_train_synthetic} - {model_name} - Variable importances.csv'))

      permutation_importances = pd.merge(pd.DataFrame(result_df_coefficients.mean(), columns=['mean']),
                          pd.DataFrame(result_df_coefficients.std(), columns=['sd']),
                          left_index=True,
                          right_index=True)

      permutation_importances.sort_values(by='mean', ascending=False).to_csv(
        os.path.join(filepath2_base,
                  f'{filepath2_train_synthetic} - {model_name} - Variable importances - Mean_{output_file}.csv'))

      plt.figure(figsize=(6, 6))
      plt.subplot(1, 1, 1)

      plt.scatter(df_correlation.iloc[:, 0], df_correlation.iloc[:, 1], color='navy', s=5)
```

```
        plt.xlim(15, 31)
        plt.ylim(15, 31)
        ax = plt.gca()
        ax.set_aspect('equal')
        plt.title(output_file)
        plt.xlabel("Measured MMSE score")
        plt.ylabel("Predicted MMSE score")
        plt.savefig(os.path.join(filepath2_base, output_file + '.png'), dpi=600, orientation='portrait',
                transparent=False,
                pad_inches=0.0)
        # plt.show()
        plt.close()

        print()

        '''
        Repeated Hold-Out cross validation 2 - Random Forest using Synthesized data (SMOGN, GAN, and
CTGAN)
        '''
        model_name = 'RF'
        N_Iterations = 1  # For k-fold CV (Set to 1 for Hold-Out validation)

        # # Grid Search before each validation
        # model = RandomForestRegressor(random_state=seed)
        # param_grid = {
        #     'n_estimators': [200, 300, 400],  # Number of trees to test
        #     'max_depth': [5, 10, 20],  # Maximum depth to test
        #     'min_samples_split': [2, 5, 8],  # Minimum number of samples required to split an internal node
        #     'min_samples_leaf': [2, 4, 6],  # Minimum number of samples required to be at a leaf node
        #     'max_features': ['sqrt']
        # }
        # grid_search = GridSearchCV(estimator=model, param_grid=param_grid, cv=5, n_jobs=-1, verbose=2)

        for k in range(N_Iterations):
            print(model_name + " - Hold-Out", str(k), " ---------------------------------------------")

            result_dfs = []  # To store results for each iteration
            result_coefficient_dfs = []
            model_coefficients_dfs = []

            x_train = train_dataset[COLUMNS_VARIABLES]
            scaler = sklearn.preprocessing.StandardScaler()
            x_train_norm = pd.DataFrame(scaler.fit_transform(x_train), columns=x_train.columns)
            x_train_norm_values = x_train_norm.values

            y_train = train_dataset[COLUMNS_RESPONSE].values.ravel()
            x_test = test_dataset[COLUMNS_VARIABLES]
            x_test_norm = pd.DataFrame(scaler.fit_transform(x_test), columns=x_test.columns)
            x_test_norm_values = x_test_norm.values
            y_test = test_dataset[COLUMNS_RESPONSE].values.ravel()

            # # If Grid search: Re-fit using the best parameters
            # grid_search.fit(x_train_norm, y_train)
            # best_params = grid_search.best_params_
            # model = RandomForestRegressor(random_state=seed, **best_params)
            model = RandomForestRegressor(random_state=seed, n_estimators=300, max_depth=10,
min_samples_split=5, min_samples_leaf=4, max_features='sqrt')
```

```
            # Training
            model.fit(x_train_norm, y_train)

            y_pred = model.predict(x_test_norm)
            # Add DataFrame to the list
            df = pd.merge(pd.DataFrame(y_test), pd.DataFrame(y_pred), left_index=True, right_index=True)
            df = pd.merge(pd.DataFrame(df), pd.DataFrame(x_test).reset_index(drop=True), left_index=True,
                    right_index=True)
            result_dfs.append(df)

            # Calculate MAE, RMSE, and COR by comparing predictions and actual values
            mae = np.mean(np.abs(df.iloc[:, 1] - df.iloc[:, 0]))
            rmse = np.sqrt(mean_squared_error(df.iloc[:, 0], df.iloc[:, 1]))
            cor = df.corr().iloc[0, 1]

            # Save results to a CSV file
            df.to_csv(
              os.path.join(filepath2_base,
                      f'{filepath2_train_synthetic} - {model_name} -
Round{k}_MAE{mae:.3f}_RMSE{rmse:.3f}_COR{cor:.3f}.csv'))

            # Add model parameters and feature importance to the DataFrame
            model_params = model.get_params()
            # # If Grid Search
            # model_params = best_params.copy()
            model_params.update(
              {'Feature_Importance_' + col: imp for col, imp in
               zip(COLUMNS_VARIABLES, model.feature_importances_)})
            coefficients = pd.DataFrame([model_params])
            model_coefficients_dfs.append(coefficients)
            model_coefficients_dfs[0].to_csv(
              os.path.join(filepath2_base, f'{filepath2_train_synthetic} - {model_name} - Coefficients.csv'))

            # Permutation Importance
            scores = permutation_importance(model, x_test_norm, y_test)
            result_coefficient_dfs.append(pd.DataFrame([scores], columns=x_test.columns))

            # For external tests
            x_ex_test = ex_test_dataset[COLUMNS_VARIABLES]
            x_ex_test_norm = pd.DataFrame(scaler.fit_transform(x_ex_test), columns=x_ex_test.columns)
            x_ex_test_norm_values = x_ex_test_norm.values

            y_ex_test = ex_test_dataset[COLUMNS_RESPONSE]

            y_ex_pred = model.predict(x_ex_test_norm)

            df_ex = pd.merge(pd.DataFrame(y_ex_test.values), pd.DataFrame(y_ex_pred), left_index=True,
                      right_index=True)
            df_ex = pd.merge(pd.DataFrame(df_ex), pd.DataFrame(x_ex_test).reset_index(drop=True),
                      left_index=True,
                      right_index=True)

            mae = np.mean(np.abs(df_ex.iloc[:, 1] - df_ex.iloc[:, 0]))
            rmse = np.sqrt(mean_squared_error(df_ex.iloc[:, 0], df_ex.iloc[:, 1]))
            cor = df_ex.corr().iloc[0, 1]

            df_ex.to_csv(
              os.path.join(filepath2_base,
```

```
                            f'{filepath2_train_synthetic} - {model_name} - External Test -
Round{k}_MAE{mae:.3f}_RMSE{rmse:.3f}_COR{cor:.3f}.csv'))

        # Combine DataFrames in the list
        df_correlation = pd.concat(result_dfs, ignore_index=True)
        result_df_coefficients = pd.concat(result_coefficient_dfs, ignore_index=True)

        # Calculate MAE, MSE, and COR for the combined results DataFrame
        total_mae = np.mean(np.abs(df_correlation.iloc[:, 1] - df_correlation.iloc[:, 0]))
        total_rmse = np.sqrt(mean_squared_error(df_correlation.iloc[:, 0], df_correlation.iloc[:, 1]))
        total_cor = df_correlation.corr().iloc[0, 1]

        output_file = f'{filepath2_train_synthetic} - {model_name} - Repeated Hold-Out Validation - {Iter_SEED} -
MAE{total_mae:.3f}_RMSE{total_rmse:.3f}_COR{total_cor:.3f}'
        print(output_file)
        df_correlation.to_csv(os.path.join(filepath2_base, f'{output_file}.csv'))

        # Combine coefficients DataFrames
        model_coefficients_df = pd.concat(model_coefficients_dfs, ignore_index=True)
        model_coefficients_df.to_csv(
            os.path.join(filepath2_base,
                         f'{filepath2_train_synthetic} - {model_name} - Coefficients_Round_{k}.csv'))

        result_df_coefficients.to_csv(
            os.path.join(filepath2_base,
                         f'{filepath2_train_synthetic} - {model_name} - Variable importances.csv'))

        permutation_importances = pd.merge(pd.DataFrame(result_df_coefficients.mean(), columns=['mean']),
                                           pd.DataFrame(result_df_coefficients.std(), columns=['sd']),
                                           left_index=True,
                                           right_index=True)

        permutation_importances.sort_values(by='mean', ascending=False).to_csv(
            os.path.join(filepath2_base,
                         f'{filepath2_train_synthetic} - {model_name} - Variable importances - Mean_output_file.csv'))

        plt.figure(figsize=(6, 6))
        plt.subplot(1, 1, 1)

        plt.scatter(df_correlation.iloc[:, 0], df_correlation.iloc[:, 1], color='navy', s=5)
        plt.xlim(15, 31)
        plt.ylim(15, 31)
        ax = plt.gca()
        ax.set_aspect('equal')
        plt.title(output_file)
        plt.xlabel("Measured MMSE score")
        plt.ylabel("Predicted MMSE score")
        plt.savefig(os.path.join(filepath2_base, output_file + '.png'), dpi=600, orientation='portrait',
                    transparent=False,
                    pad_inches=0.0)
        # plt.show()
        plt.close()

        print()

        '''
        Repeated Hold-Out validation 3 - DNN using Synthesized data
        '''
```

```
Num_Classes = 2
model_name = 'DNN'
N_Iterations = 1  # For k-fold CV (Set to 1 for Hold-Out validation)

for k in range(N_Iterations):
    print(model_name + " - Hold-Out", str(k), " --------------------------------------------")

    result_dfs = []  # List to store results
    result_coefficient_dfs = []  # List to store coefficients
    model_coefficients_dfs = []

    x_train = train_dataset[COLUMNS_VARIABLES]
    scaler = sklearn.preprocessing.StandardScaler()
    x_train_norm_values = scaler.fit_transform(x_train)
    y_train = train_dataset[COLUMNS_RESPONSE].values.ravel()
    x_test = test_dataset[COLUMNS_VARIABLES]
    x_test_norm_values = scaler.transform(x_test)
    y_test = test_dataset[COLUMNS_RESPONSE].values.ravel()

    # Training
    model = getModel(x_train_norm_values, Num_Classes)
    model, history = trainModel(model, x_train_norm_values, y_train, best_only_mode=True)
    # model, history = trainModel(model, x_train_norm_values, y_train, best_only_mode=True)

    y_pred = model.predict(x_test_norm_values, verbose=0)
    # Add DataFrame to the list
    df = pd.merge(pd.DataFrame(y_test), pd.DataFrame(y_pred), left_index=True, right_index=True)
    df = pd.merge(pd.DataFrame(df), pd.DataFrame(x_test).reset_index(drop=True), left_index=True,
            right_index=True)
    result_dfs.append(df)

    # Calculate MAE, RMSE, and COR by comparing predictions and actual values
    mae = np.mean(np.abs(df.iloc[:, 1] - df.iloc[:, 0]))
    rmse = np.sqrt(mean_squared_error(df.iloc[:, 0], df.iloc[:, 1]))
    cor = df.corr().iloc[0, 1]

    # Save results to a CSV file
    df.to_csv(
        os.path.join(filepath2_base,
                f'{filepath2_train_synthetic} - {model_name} - Round{k}-
{j}_MAE{mae:.3f}_RMSE{rmse:.3f}_COR{cor:.3f}.csv'))

    pd.DataFrame(history.history).to_csv(
        os.path.join(filepath2_base,
                f'{filepath2_train_synthetic} - {model_name} - History - Round{k}-
{j}_MAE{mae:.3f}_RMSE{rmse:.3f}_COR{cor:.3f}.csv'))

    # Plot training history
    plt.figure(figsize=(12, 4))
    plt.subplot(1, 2, 1)
    plt.plot(history.history['loss'], label='loss')
    plt.plot(history.history['val_loss'], label='val_loss')
    # plt.plot(history.history['val_loss'], label='val_loss')
    plt.ylim(0, 20)
    plt.title('Training Loss and Validation Loss')
    plt.xlabel('Epochs')
    plt.ylabel('Loss')
    plt.legend()
```

```
            plt.subplot(1, 2, 2)
            plt.plot(history.history['mae'], label='MAE')
            plt.plot(history.history['r2_score'], label='R2 Score')
            plt.ylim(-2, 3)
            plt.title('MAE and R2 Score')
            plt.xlabel('Epochs')
            plt.ylabel('Metric')
            plt.legend()

            plt.tight_layout()
            # plt.show()
            plt.savefig(os.path.join(filepath2_base,
                                     f'{filepath2_train_synthetic} - {model_name} - History - Round{k}-
{j}_MAE{mae:.3f}_RMSE{rmse:.3f}_COR{cor:.3f}.png'),
                        format='png')
            # plt.close()

            # Permutation Importance
            scores = permutation_importance(model, x_test_norm_values, y_test)
            result_coefficient_dfs.append(pd.DataFrame([scores], columns=x_test.columns))

            # For external tests
            x_ex_test = ex_test_dataset[COLUMNS_VARIABLES]
            x_ex_test_norm_values = scaler.transform(x_ex_test)
            y_ex_test = ex_test_dataset[COLUMNS_RESPONSE]

            y_ex_pred = model.predict(x_ex_test_norm_values, verbose=0)

            df_ex = pd.merge(pd.DataFrame(y_ex_test.values), pd.DataFrame(y_ex_pred), left_index=True,
                             right_index=True)
            df_ex = pd.merge(pd.DataFrame(df_ex), pd.DataFrame(x_ex_test).reset_index(drop=True),
                             left_index=True,
                             right_index=True)

            mae = np.mean(np.abs(df_ex.iloc[:, 1] - df_ex.iloc[:, 0]))
            rmse = np.sqrt(mean_squared_error(df_ex.iloc[:, 0], df_ex.iloc[:, 1]))
            cor = df_ex.corr().iloc[0, 1]

            df_ex.to_csv(
                os.path.join(filepath2_base,
                             f'{filepath2_train_synthetic} - {model_name} - External Test - Round{k}-
{j}_MAE{mae:.3f}_RMSE{rmse:.3f}_COR{cor:.3f}.csv'))

        # Combine DataFrames in the list
        result_df = pd.concat(result_dfs, ignore_index=True)
        result_df_coefficients = pd.concat(result_coefficient_dfs, ignore_index=True)

        # Calculate MAE, MSE, and COR for the combined results DataFrame
        total_mae = np.mean(np.abs(result_df.iloc[:, 1] - result_df.iloc[:, 0]))
        total_rmse = np.sqrt(mean_squared_error(result_df.iloc[:, 0], result_df.iloc[:, 1]))
        total_cor = result_df.corr().iloc[0, 1]

        print(
            f'{filepath2_train_synthetic} - {model_name} - Hold-Out - {Iter_SEED} - Round{k}
MAE{total_mae:.3f}_RMSE{total_rmse:.3f}_COR{total_cor:.3f}')
        result_df.to_csv(os.path.join(filepath2_base,
```

```
                    f'{filepath2_train_synthetic} - {model_name} - Hold-Out - {Iter_SEED} - Round{k}
MAE{total_mae:.3f}_RMSE{total_rmse:.3f}_COR{total_cor:.3f}.csv'))

        result_df_coefficients.to_csv(
          os.path.join(filepath2_base,
                 f'{filepath2_train_synthetic} - {model_name} - Variable importances.csv'))

        permutation_importances = pd.merge(pd.DataFrame(result_df_coefficients.mean(), columns=['mean']),
                             pd.DataFrame(result_df_coefficients.std(), columns=['sd']),
                             left_index=True,
                             right_index=True)

        permutation_importances.sort_values(by='mean', ascending=False).to_csv(
          os.path.join(filepath2_base,
                 f'{filepath2_train_synthetic} - {model_name} - Variable importances - Mean - {Iter_SEED} -
Round{k} MAE{total_mae:.3f}_RMSE{total_rmse:.3f}_COR{total_cor:.3f}.csv'))

        # plt.figure(figsize=(12, 4))
        plt.figure(figsize=(6, 6))
        plt.subplot(1, 1, 1)

        plt.scatter(result_df.iloc[:, 0], result_df.iloc[:, 1], color='navy', s=5)
        plt.xlim(15, 31)
        plt.ylim(15, 31)
        ax = plt.gca()
        ax.set_aspect('equal')
        plt.title(f'{model_name} - MAE{total_mae:.3f}_RMSE{total_rmse:.3f}_COR{total_cor:.3f}')
        plt.xlabel("Measured MMSE score")
        plt.ylabel("Predicted MMSE score")
        plt.savefig(os.path.join(filepath2_base,
                    f'{filepath2_train_synthetic} - {model_name} - Hold-Out - {Iter_SEED} - Round{k}
MAE{total_mae:.3f}_RMSE{total_rmse:.3f}_COR{total_cor:.3f}.png'),
              dpi=600, orientation='portrait',
              transparent=False,
              pad_inches=0.0)

        print()
```
